# Steroid hormone catabolites activate the pyrin inflammasome through a non-canonical mechanism

**DOI:** 10.1016/j.celrep.2022.111472

**Published:** 2022-10-11

**Authors:** Flora Magnotti, Daria Chirita, Sarah Dalmon, Amandine Martin, Pauline Bronnec, Jeremy Sousa, Olivier Helynck, Wonyong Lee, Daniel L. Kastner, Jae Jin Chae, Michael F. McDermott, Alexandre Belot, Michel Popoff, Pascal Sève, Sophie Georgin-Lavialle, Hélène Munier-Lehmann, Tu Anh Tran, Ellen De Langhe, Carine Wouters, Yvan Jamilloux, Thomas Henry

**Affiliations:** 1 CIRI, Centre International de Recherche en Infectiologie, Inserm U1111, Université Claude Bernard Lyon 1, CNRS, UMR5308, ENS de Lyon, University Lyon, 69007 Lyon, France; 2 Institut Pasteur, Université de Paris Cité, CNRS UMR3523, Chemistry and Biocatalysis Unit, 75724 Paris Cedex 15, France; 3 Inflammatory Disease Section, Metabolic, Cardiovascular and Inflammatory Disease Genomics Branch, National Human Genome Research Institute, Bethesda, MD, USA; 4 Leeds Institute of Rheumatic and Musculoskeletal Medicine, St James’s University Hospital, Leeds, UK; 5 Department of Pediatric Nephrology, Rheumatology, Dermatology, Reference Centre for Rheumatic, AutoImmune and Systemic Diseases in Children (RAISE), Hôpital Femme Mère Enfant, CHU Lyon, Lyon, France; 6 LIFE, Lyon Immunopathology Federation, Lyon, France; 7 Bacterial Toxins, Institut Pasteur, Paris, France; 8 Department of Internal Medicine, University Hospital Croix-Rousse, Lyon 1 University, Lyon, France; 9 Sorbonne University, Department of Internal Medicine, Tenon Hospital, DMU 3ID, AP-HP, National Reference Center for Autoinflammatory Diseases and Inflammatory Amyloidosis (CEREMAIA), INSERM U938, Paris, France; 10 Department of Pediatrics, Carémeau Hospital, CHU Nîmes, Nîmes, France; 11 Division of Rheumatology, University Hospitals Leuven, Leuven, Belgium; 12 Laboratory of Tissue Homeostasis and Disease, Department of Development and Regeneration, KU Leuven, Leuven, Belgium; 13 KU Leuven—University of Leuven, Department of Microbiology and Immunology, Laboratory of Adaptive Immunology & Immunobiology, Leuven, Belgium; 14 Department of Pediatrics, University Hospitals Leuven, 3000 Leuven, Belgium; 15 These authors contributed equally; 16 Senior author; 17 Lead contact

## Abstract

The pyrin inflammasome acts as a guard of RhoA GTPases and is central to immune defenses against RhoA-manipulating pathogens. Pyrin activation proceeds in two steps. Yet, the second step is still poorly understood. Using cells constitutively activated for the pyrin step 1, a chemical screen identifies etiocholanolone and pregnanolone, two catabolites of testosterone and progesterone, acting at low concentrations as specific step 2 activators. High concentrations of these metabolites fully and rapidly activate pyrin, in a human specific, B30.2 domain-dependent manner and without inhibiting RhoA. Mutations in *MEFV*, encoding pyrin, cause two distinct autoinflammatory diseases pyrin-associated autoinflammation with neutrophilic dermatosis (PAAND) and familial Mediterranean fever (FMF). Monocytes from PAAND patients, and to a lower extent from FMF patients, display increased responses to these metabolites. This study identifies an unconventional pyrin activation mechanism, indicates that endogenous steroid catabolites can drive autoinflammation, through the pyrin inflammasome, and explains the “steroid fever” described in the late 1950s upon steroid injection in humans.

## In brief

Magnotti et al. use a chemical screen to identify pyrin inflammasome activators acting primarily on pyrin step 2. Pregnanolone and etiocholanolone, two catabolites of progesterone and testosterone, activate human pyrin in a B30.2-dependent manner. Pyrin-mutated PAAND patients are highly responsive to pregnanolone. These endogenous catabolites could contribute to sterile (auto)inflammation.

## Graphical Abstract

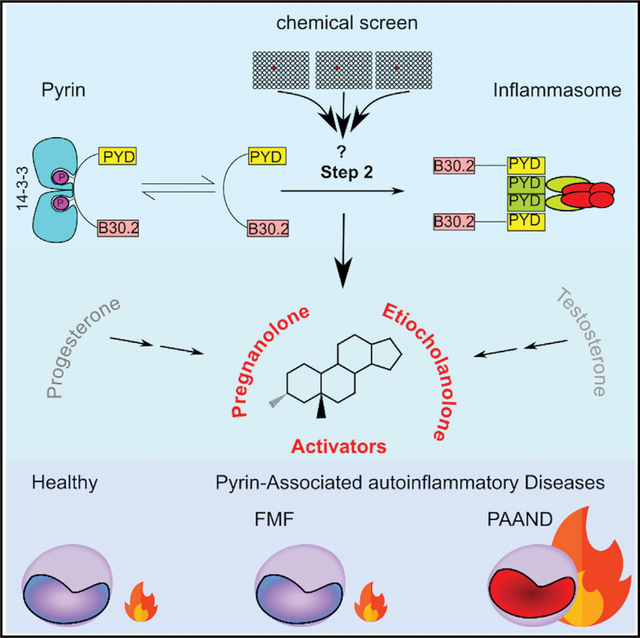

## INTRODUCTION

Inflammasomes are innate immune complexes that contribute to antimicrobial responses (Broz and Monack, 2011), but can also be deleterious in various chronic autoinflammatory conditions (Peckham et al., 2017). Inflammasome activation results in activation of the inflammatory caspase-1, cleavage of the poreforming protein GasderminD (GSDMD) that triggers a fast cell death, termed pyroptosis, and release of the inflammatory cytokines interleukin-1β (IL-1β) and IL-18. Inflammasome sensors can act as direct pathogen-associated molecular pattern receptors but have also evolved more general sensing mechanisms allowing them to be activated in response to damage-associated molecular patterns or to homeostasis-altering molecular processes (Liston and Masters, 2017). Furthermore, the NLRP3 inflammasome is regulated by several metabolites (Hughes and O’Neill, 2018). Whether metabolites regulate other inflammasome sensors is currently unknown.

**Figure 1.**
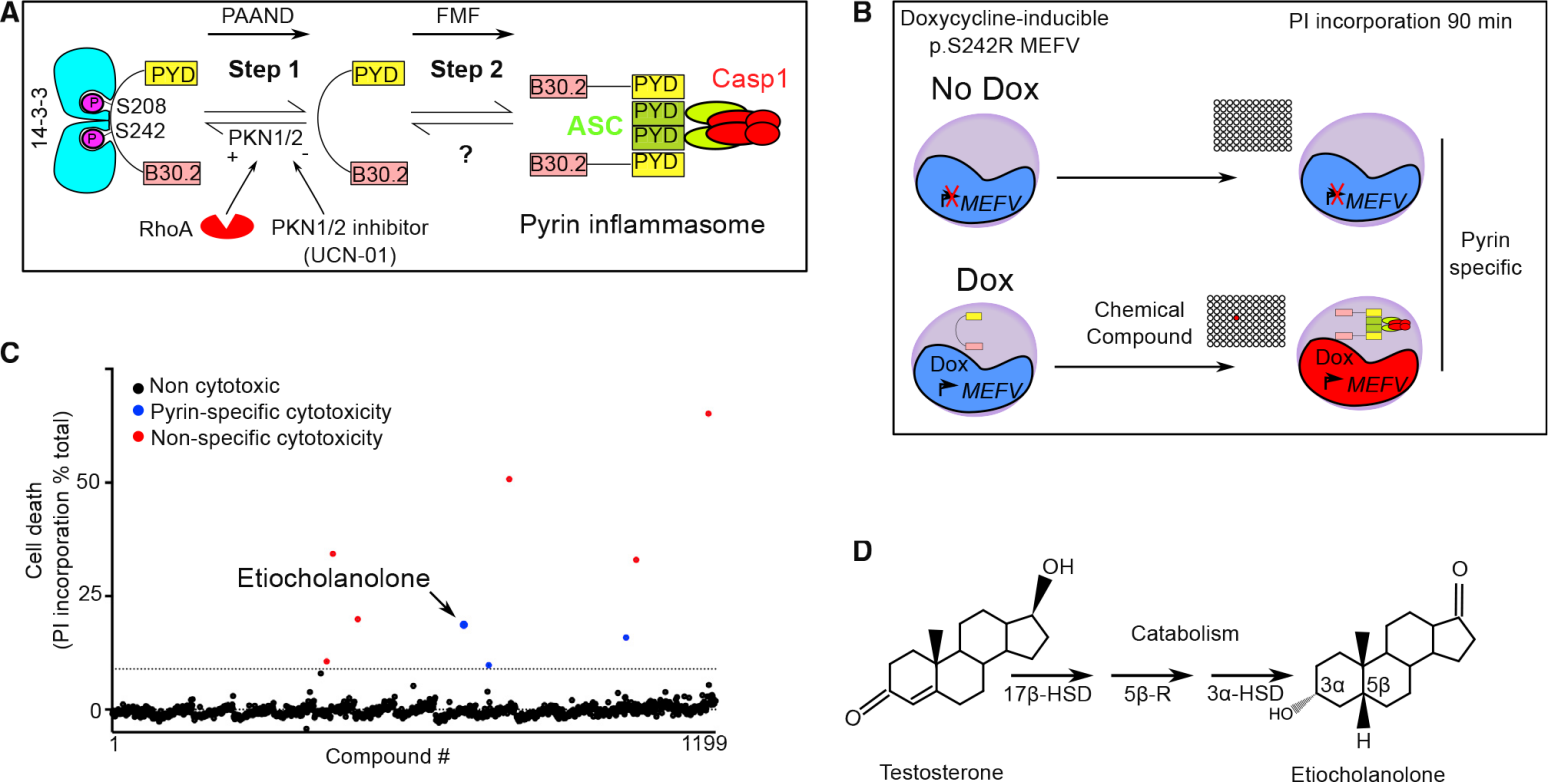
A chemical screen identifies etiocholanolone, a testosterone catabolite, as a pyrin inflammasome step 2 activator (A) Model for pyrin two-step activation mechanism. Step 1 is due to dephosphorylation of pyrin and loss of 14–3-3 binding and is constitutive in PAAND patients. Step 2 is uncharacterized but is upstream of ASC speck formation and is constitutive in FMF patients. (B) Chemical screen overview: cells expressing doxycycline (Dox) or not (No Dox) p.S242R *MEFV* were exposed to individual chemical compounds. Ninety-minute post-exposure, cell death was monitored using propidium iodide (PI). Compounds driving cell death independently of pyrin (i.e., in the absence of Dox) were excluded. (C) Screen results are shown, each dot represents the cell death value of cells exposed to one chemical compound. The dotted line represents the mean + 3 SD. Red dots represent non-specific hits (killing cells irrespective of the presence or the absence of Dox) while blue dots represent specific hits displaying cytotoxicity only upon pyrin expression. Etiocholanolone (Etio) (6.9 μM) is highlighted. Each chemical compound was screened once in the presence and in the absence of Dox. The value shown corresponds to normalized cell death value of a single well. (D) Structure of progesterone and its catabolite etiocholanolone are shown. The stereochemistry of carbon 3 and 5 is indicated. 17β-HSD, 17β-hydroxy-steroid dehydrogenase; 5β-R, 5β-reductase; 3α-HSD, 3α-hydroxy-steroid dehydrogenase.

Pyrin is an inflammasome sensor acting as a guard of Rho GTPase activity (Xu et al., 2014). Bacterial toxins (e.g., *Clostridioides difficile* toxins A and B [TcdA/B]) or bacterial effectors (e.g., *Yersinia* YopE/T [Chung et al., 2016; Ratner et al., 2016]) inhibit RhoA and trigger pyrin inflammasome activation. Similarly, by disrupting RhoA prenylation, mevalonate kinase deficiency activates pyrin (Akula et al., 2016). RhoA inhibition lifts the dynamic blockage of pyrin. Indeed, at steady state, pyrin is phosphorylated by PKN1/2 on two serine residues (S208 and S242), allowing a phospho-dependent chaperone from the 14–3-3 family to sequester pyrin away from downstream inflammasome molecules. PKN1/2, two kinases from the PKC superfamily, are RhoA effectors that are active in cells with homeostatic levels of RhoA activation. Inhibition of RhoA leads to loss of PKN1/2 activity, dephosphorylation of pyrin, and ultimately triggers activation of the pyrin inflammasome (Akula et al., 2016; Gao et al., 2016; Masters et al., 2016; Xu et al., 2014). Yet, pyrin dephosphorylation is not sufficient to trigger the full pyrin inflammasome activation (Magnotti et al., 2019). We have therefore proposed a two-step model for pyrin activation (Figure 1A). The first step corresponds to pyrin dephosphorylation, while the second step, which remains poorly understood, corresponds to the formation of ASC oligomers and caspase-1 activation. Interestingly, this model is consistent with the observation that colchicine, and other microtubule-depolymerizing drugs, block pyrin activation downstream of its dephosphorylation (Gao et al., 2016; Van Gorp et al., 2016).

The two-step model is also supported by the two distinct autoinflammatory diseases associated with mutations in *MEFV*, the gene encoding pyrin (Jamilloux et al., 2018). Indeed, mutations in *MEFV* exon 10, coding the B30.2 domain of pyrin, cause familial Mediterranean fever (FMF) and are associated with a constitutively activated step 2 (Magnotti et al., 2019). In contrast, mutations affecting the serine residues, which are dephosphorylated during step 1 (Hong et al., 2019; Masters et al., 2016), or the neighboring residues required for the interaction with 14–3-3 proteins (Moghaddas et al., 2017), cause pyrin-associated autoinflammation with neutrophilic dermatosis (PAAND).

Despite its importance in health and disease, the mechanism responsible for step 2 is still largely unclear and this study aimed at increasing our knowledge on this specific regulatory mechanism.

## RESULTS

### A chemical screen identified sex hormone catabolites as pyrin step 2 activators

To identify molecules that trigger step 2 of the pyrin inflammasome cascade, we used a human monocytic cell line (U937) expressing the PAAND *MEFV* variant (i.e., p.S242R) under the control of a doxycycline-inducible promoter (Magnotti et al., 2019) (Figure 1B). In these cells, the p.S242R mutation mimics pyrin dephosphorylation resulting in a pyrin protein constitutively activated for step 1. Cells expressing (in the presence of doxycycline) p.S242R *MEFV* were treated with compounds (n = 1,199) from the Prestwick “FDA-approved” chemical library (see STAR methods and Table S2 for details). At 90 min post-addition, cell death was measured by quantifying propidium iodide incorporation. A counter-screen was performed simultaneously under the same conditions but in the absence of doxycycline (i.e., in the absence of pyrin expression) to retain compounds triggering pyrin-specific cell death. One compound, etiocholanolone (6.9 μM), was identified as triggering a fast cell death in a doxycycline-dependent manner (Figure 1C). Etiocholanolone, also termed 3α-hydroxy 5β-androstan-17-one, is an endogenous catabolite of the steroid hormone, testosterone (Figure 1D). Except for medrysone, which was weakly active, none of the other steroids in the Prestwick library triggered pyrin-specific cell death.

**Figure 2.**
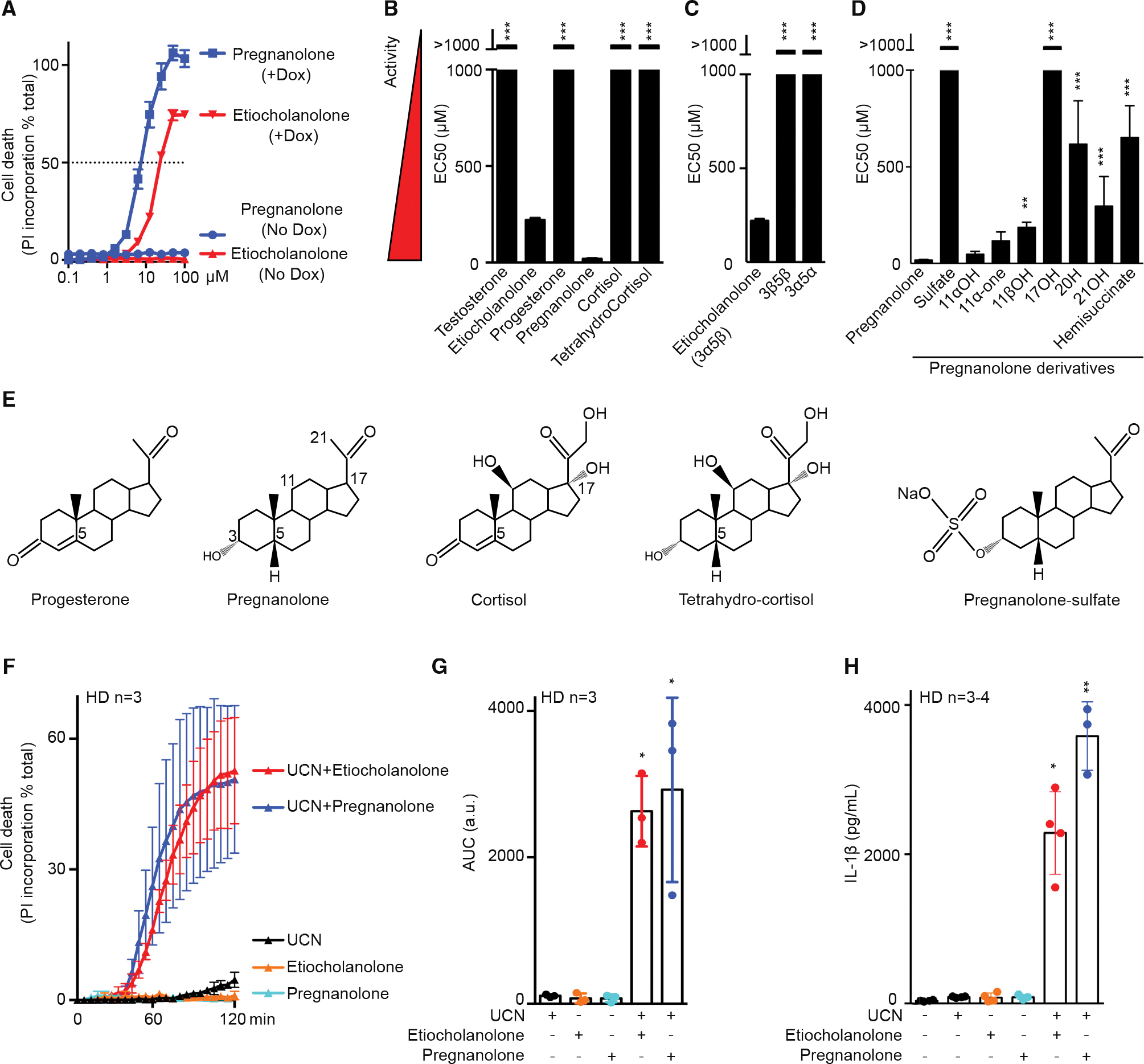
Pregnanolone and etiocholanolone specifically trigger pyrin inflammasome step 2 (A) Pregnanolone (blue) and etiocholanolone (red) were added at different concentrations on cells expressing (in the presence of doxycycline, +Dox) or not (No Dox) p.S242R *MEFV*. Cell death was determined at 3 h post-addition. The concentration triggering 50% cell death (horizontal dotted line) determined the EC_50_ (half-maximal effective concentration, which is inversely correlated to the activity of the tested molecule). (B–D) (B) Structure activity of various steroid hormones, their catabolites, (C) of etiocholanolone (also known as 3α-hydroxy 5β-androstan-17-one (3α,5β)) and its two stereoisomers 3β-hydroxy-5β-androstan-17-one (3β,5β) and androsterone (3α,5α), (D) of pregnanolone and molecules closely related. EC_50_ values were calculated as in (A). (E) Structures of selected compounds. All compounds can be found in Figure S1. (F) Primary monocytes from healthy donors (HDs, n = 3) were pre-treated with pregnanolone (6 μM) or etiocholanolone (12 μM) for 1 h followed by addition of the PKC superfamily inhibitor, UCN-01. Cell death was monitored every 5 min for 2 h. (G) The area under the curve (AUC) was computed for each HD. (H) Primary monocytes from HDs (n = 3–4) were treated with LPS for 2 h, then pre-treated with pregnanolone (6 μM) or etiocholanolone (12 μM) for 1 h followed by UCN-01 addition. IL-1β concentration in the supernatant was quantified at 3 h post-UCN-01 addition. One experiment representative of three (A) or two (B–D) independent experiment is shown. Mean and SEM of triplicates are shown. Sulfate, 5β-pregnan-3α-ol-20-one sulfate; 11αOH, 5β-pregnan-3α,11α-diol-20-one; 11-one, 5β-pregnan-3α-ol-11,20-dione; 11βOH, 5-β-pregnan-3-α,11β-diol-20-one; 17OH, 5-β-pregnan-3-α,17-diol-20-one; 20H, 5-β-pregnan-3-α-ol; 21OH, 5-β-pregnan-3-α,21-diol-20-one; hemisuccinate, 5-β-pregnan-3-α,21-diol-20-one 21 hemisuccinate. (B–D) One-way ANOVA with Dunn’s correction was applied. ***p < 0.001, **p = 0.007. (F) Each point corresponds to the mean ± SEM of three HD values, each one being the mean of a triplicate. (F) Each point corresponds to the mean ± SEM of three HD values, each one being the mean of a triplicate. (G) Each point corresponds to the mean AUC of kinetics of one HD performed in triplicate, the bar represents the mean ± SEM of three HD values. AUC are expressed as arbitrary units (a.u.). Friedman test with Dunn’s correction for multiple analysis was performed in comparison with untreated cells. *p = 0.023 (Etio + UCN); p = 0.011 (Pregna + UCN). (H) Each point corresponds to the mean IL-1β concentration of one HD calculated from a triplicate, the bar represents the mean ± SEM of three to four HDs. Kruskal-Wallis test with Dunn’s correction for multiple analysis was performed in comparison with LPS-treated cells. *p = 0.011, **p = 0.002.

The ability of etiocholanolone to trigger cell death in a p.S242R-*MEFV*-dependent manner was validated in an independent set of experiments and demonstrated to be dose dependent (Figure 2A). To assess the specificity of etiocholanolone and perform structure-activity relationship studies, a number of hormones and catabolites were then tested. Interestingly, pregnanolone (3α-hydroxy-5β-pregnan-20-one), a catabolite of progesterone, triggered p.S242R-*MEFV*-dependent cell death at an even lower concentration than etiocholanolone (Figure 2A). In contrast, neither testosterone nor progesterone triggered cell death, even at high concentrations (half-maximal effective concentration [EC_50_] > 1,000 μM), and despite similar lipophilic properties as etiocholanolone and pregnanolone (Figure S1). Furthermore, both cortisol and its catabolite, tetrahydrocortisol, were inactive (Figure 2B), demonstrating the specificity of the pregnanolone and etiocholanolone in triggering this response.

Etiocholanolone and pregnanolone share the same stereochemistry on carbons 3 (3α) and 5 (5β) of the sterol. To evaluate the stereospecificity of the response, the two stereoisomers of etiocholanolone were tested for their ability to trigger p.S242R-*MEFV*-dependent cell death. 3β-Hydroxy-5β-androstan-17-one (3β,5β) and androsterone (i.e., 3α-hydroxy 5α-androstan-17-one [3α,5α]), displayed EC_50_ values greater than 1,000 μM (Figures 2C and S1), indicating that the response is stereospecific.

Furthermore, experiments with pregnanolone derivatives demonstrated that sulfation, a modification associated with steroid inactivation and excretion (Schiffer et al., 2019), leads to a complete loss of activity (Figure 2D). All the modifications tested on the sterol ring or the terminal carbon or ketone (Figure S1) decreased the pyrin-specific cytotoxicity. In particular, hydroxylation on C17 fully abolished the cell death, thereby explaining the absence of activity of the cortisol catabolite (Figures 2D and 2E). Overall, these structure-function analyses demonstrated that etiocholanolone and pregnanolone are highly specific compounds that trigger S242R-pyrin-mediated cell death.

To validate this result in primary human monocytes, we used the PKC superfamily inhibitor, UCN-01, which inhibits PKN1/2 and dephosphorylates pyrin, thus recapitulating the effect of the p.S242R mutation. As described previously (Magnotti et al., 2019, 2020), UCN-01 alone does not trigger pyroptosis in primary human monocytes from healthy donors (HDs). Similarly, neither etiocholanolone (12 μM) nor pregnanolone (6 μM) alone were cytotoxic, even after long incubation times (Figure S2A). Yet, the combination of UCN-01 and either of these two steroid catabolites triggered a very fast cell death (Figures 2F and 2G) and IL-1β release (Figure 2H). These results thus strongly suggest that, in primary monocytes, steroid catabolites activate pyrin step 2 and trigger activation of the pyrin inflammasome and pyroptosis in the presence of the step 1 activator, UCN-01.

### High concentrations of steroid catabolites trigger full activation of the pyrin inflammasome in the absence of a step 1 activator

**Figure 3.**
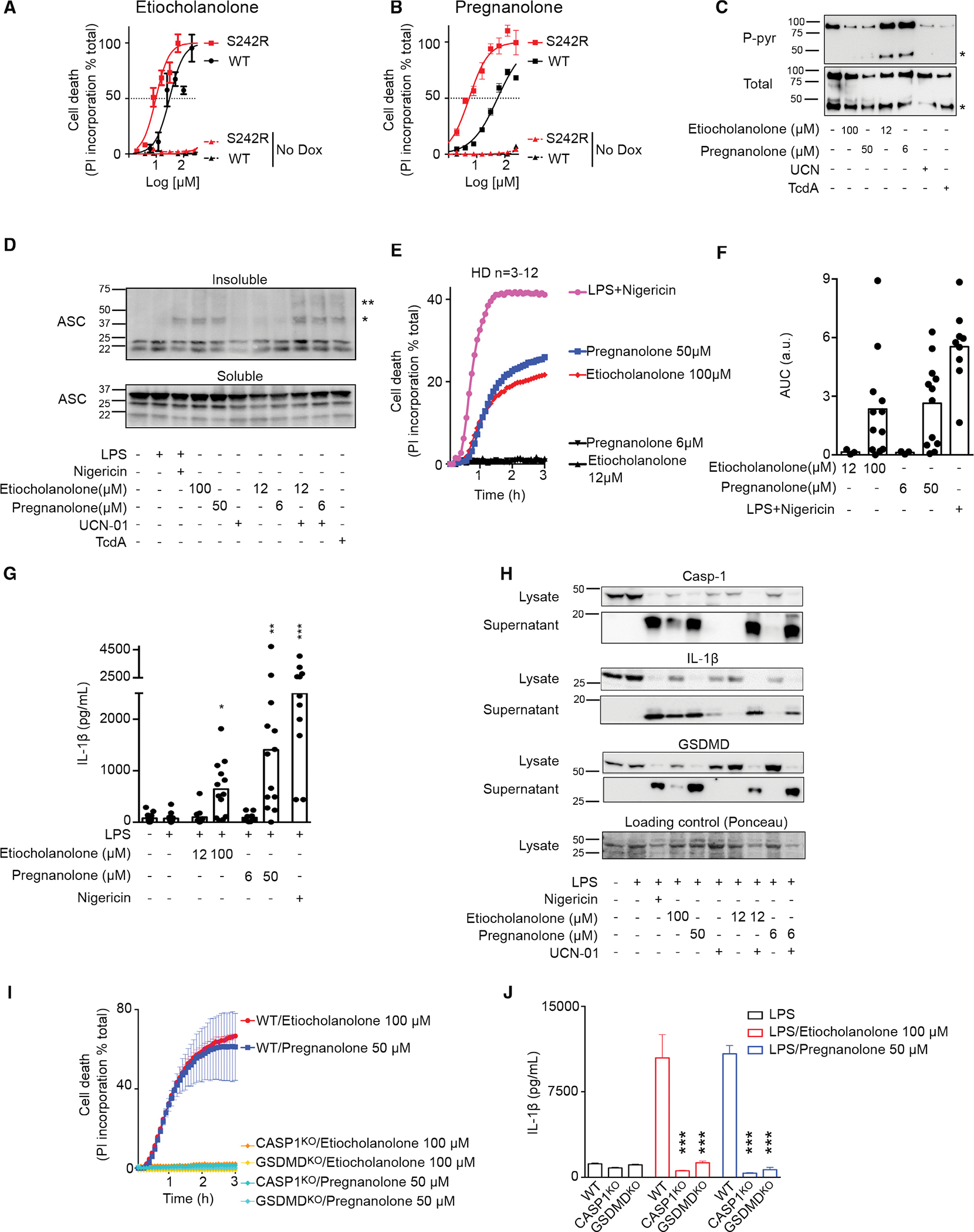
High concentrations of etiocholanolone and pregnanolone trigger full activation of pyrin inflammasome (A and B) U937 cells expressing Dox (plain lines) or not (No Dox, dotted lines) p.S242R (red) or WT (black) *MEFV* were treated with various concentration of etiocholanolone (A) or pregnanolone (B). Cell death was determined at 3 h post-addition. (C) 3xFlag-WT pyrin from U937 cells treated with the indicated stimuli at the indicated concentrations was immunoprecipitated. Ser242 phosphorylation (P-pyr) and total pyrin levels were monitored by western blot. “*” indicates a cleaved form of pyrin. (D) ASC immunoblot from U937 cells expressing WT *MEFV*. Cells were treated with the indicated molecules. ASC oligomers in the insoluble fraction were treated with DSS (2 mM) cross linker after lysis. ASC monomers in the soluble fraction is shown. “*” and “**” correspond to the sizes of ASC dimer and trimer, respectively. (E) Monocytes from HDs (n = 3–12) were treated with low (black) or high concentrations of etiocholanolone (red) and pregnanolone (blue) or with LPS + nigericin (magenta). Propidium iodide (PI) incorporation was monitored every 5 min for 3 h. (F) The corresponding area under the curve (AUC) for each donor are shown. (G) Monocytes were primed with LPS for 3 h and exposed to the indicated stimuli at the indicated concentrations. IL-1β concentration in the supernatant was quantified at 3 h post-addition. (H) Monocytes from one HD were primed or not with LPS for 3 h before addition of the indicated stimuli. Caspase-1, IL-1β, and GSDMD processing were analyzed by western blot in the cell lysate and supernatant at 3 h post-stimuli addition. (I and J) (I) *MEFV*-expressing U937 monocytes or (J) PMA-differentiated U937 macrophages WT or knockout for *CASP1* or *GSDMD* as indicated were treated with doxycycline during 16 h. (I) PI incorporation was monitored every 5 min for 3 h post stimuli addition. (J) Cells were primed with LPS for 3 h before addition of the indicated stimuli. IL-1β concentration in the supernatant was quantified at 3 h post-addition. One experiment representative of three (A–C) to two (H and I) independent experiments is shown. Mean and SEM of triplicates are shown. (A and B) Non-linear regression curve computed using least squares fit method is shown. (G) Kruskal-Wallis test with Dunn’s multiple comparisons tests was performed to compare the different treatments with the LPS treatment. *p = 0.026, **p = 0.0011, ***p < 0.001. (J) One-way ANOVA analysis with Sidak’s multiple comparisons test was performed to compare WT U937 to *CASP1*^KO^ or *GSDMD*^KO^ cells. ***p < 0.001.

Interestingly, while at low concentration (<12 μM), steroid catabolites required activation of step 1, through either genetic (p.S242R) or chemical (UCN-01) means, we noticed that, at high concentrations, both etiocholanolone (Figure 3A) and pregnanolone (Figure 3B) triggered death of U937 cells expressing WT *MEFV*. High doses of pregnanolone (50 μM) or etiocholanolone (100 μM) triggered pyrin dephosphorylation to a similar extent as TcdA or UCN-01, while low doses of steroid catabolites did not (Figure 3C). Calyculin A, a phosphatase inhibitor, was recently shown to inhibit pyrin inflammasome in response to bacterial toxins and infections (Malik et al., 2022). Similarly, Calyculin A blocked etiocholanolone- and pregnanolone-mediated cell death and IL-1β release in HD monocytes and U937 cells expressing pyrin (Figures S2B–S2D). In addition, high doses of steroid catabolites triggered ASC oligomerization, as revealed by cross-linking experiments and western blot analysis (Figure 3D). Low doses of etiocholanolone or pregnanolone did not, unless combined with UCN-01. These results suggested that, at high doses, these molecules can trigger both step 1 (i.e., pyrin dephosphorylation) and step 2 to promote ASC oligomerization and inflammasome activation. Accordingly, primary human monocytes exposed to high doses of steroid catabolites (50–100 μM) underwent a fast cell death (Figures 3E and 3F) and released IL-1β (Figure 3G). LPS priming was not required for etiocholanolone- or pregnanolone-induced monocyte death (Figures 3E and 3F), but was used to induce proIL-1β expression whenever inflammasome activation was monitored by quantifying IL-1β release by ELISA. Primary neutrophils, treated with steroid catabolites, also underwent a rapid cell death (Figure S3A). In contrast, steroid catabolites did not demonstrate substantial cytotoxicity toward lymphocytes (Figure S3B) in agreement with the lack of *MEFV* expression in these cells (Figure S3C). Etiocholanolone or pregnanolone addition triggered processing and release of caspase-1, IL-1β, and GSDMD (Figure 3H). In agreement with the greater potency of pregnanolone compared with etiocholanolone to trigger IL-1β release (Figure 3G), pregnanolone treatment led to higher caspase-1 and GSDMD cleavage than etiocholanolone treatment. Furthermore, pre-treatment with the caspase-1 inhibitor, VX765, fully abolished IL-1β release by primary human monocytes (Figure S3D). Finally, Casp1^KO^ and GSDMD^KO^ U937 expressing WT pyrin were fully resistant to steroid catabolite-mediated cell death (Figure 3I) and did not release substantial amounts of IL-1β (Figure 3J). Altogether, these experiments demonstrated that steroid catabolites specifically triggered pyrin dephosphorylation, ASC oligomerization, inflammasome activation, caspase-1-dependent, GSDMD-dependent pyroptosis, and IL-1β release. This response was independent of NLRP3 (Figure S3E).

### Steroid catabolites differ from the prototypical activator TcdA and trigger pyrin activation in a B30.2-dependent manner and in the absence of RhoA inhibition

**Figure 4.**
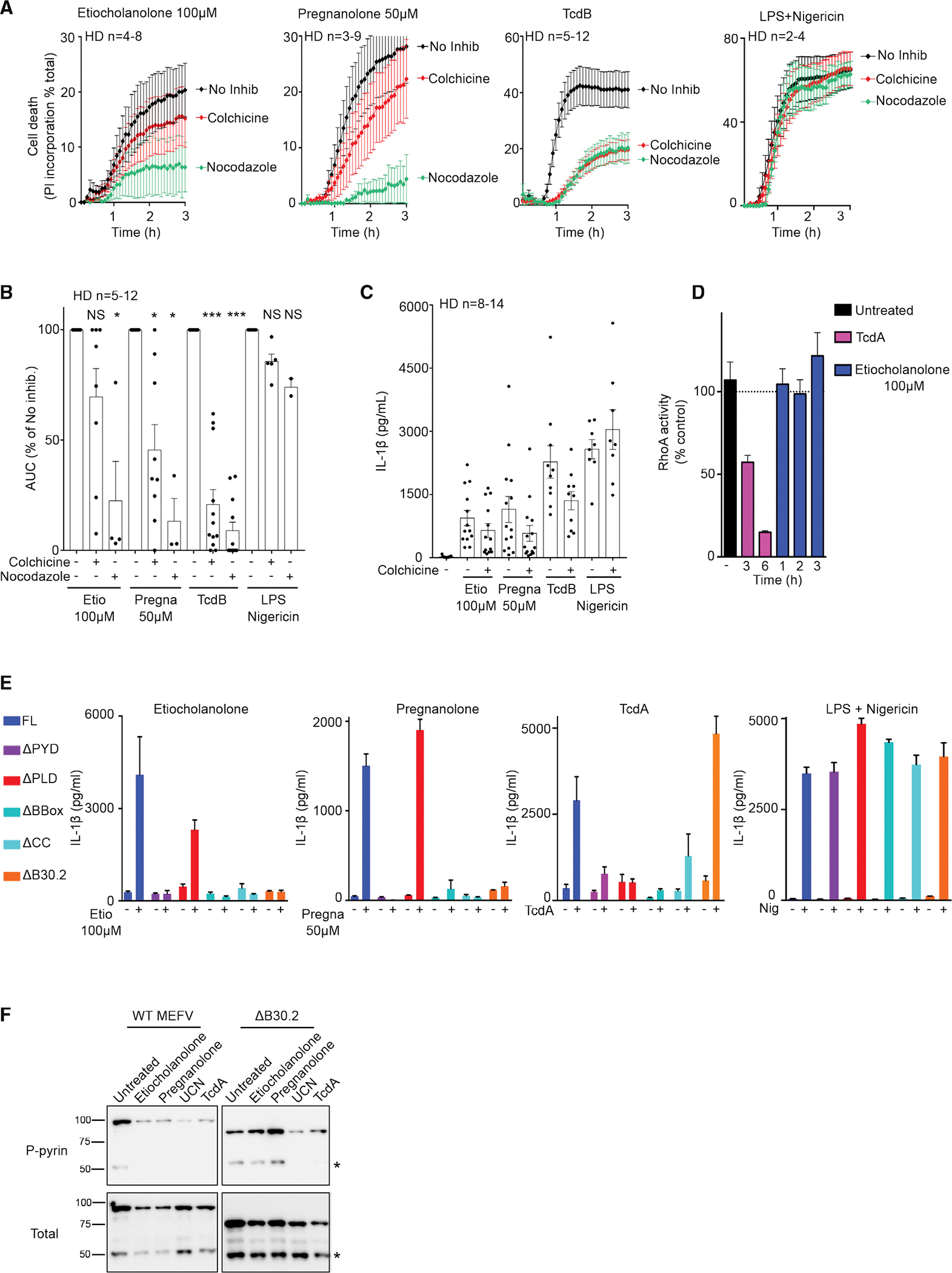
Pyrin inflammasome activation proceeds differently following TcdA/B and steroid catabolite addition (A) Monocytes from HDs (n = 2–12) were treated with colchicine or nocodazole and 30 min later with the indicated stimuli. Propidium iodide incorporation was monitored every 5 min for 3 h. (B) The area under the cure (AUC) is shown. For each HD, the AUC values in the presence of inhibitors were normalized to the AUC value obtained in the absence of inhibitor. (C) Monocytes from HDs (n = 8–14) were treated with colchicine and 30 min later with the indicated stimuli. IL-1β concentration in the supernatant was quantified at 3 h post-addition. (D) RhoA activity was determined by G-LISA at different time post-treatment in the lysate of U937 cells. The activity of the different treatments at the indicated time is presented in Figure S4B. (E) Doxycycline-induced, PMA-differentiated U937 macrophages expressing *MEFV* Full-length (FL), deleted of the pyrin (ΔPYD), of the phosphorylated linker (ΔPLD), of the BBox (ΔBbox), of the coiled-coil (ΔCC), or the B30.2 (ΔB30.2) domains were treated with LPS for 3 h and then with the indicated stimuli. IL-1β concentration in the supernatant was quantified at 3 h post-addition. (F) Doxycycline-induced, U937 monocytes expressing WT or ΔB30.2 *MEFV* were treated with the indicated stimuli for 90 min. Pyrin S242 phosphorylation was assessed by western blot analysis following immunoprecipitation. “*” indicates a cleaved form of pyrin. (A–E) Mean and SEM are shown. (B and C) Each dot represents the value for one HD. (D and E) One experiment with technical triplicates representative of two (D) to three (E) independent experiments is shown. (B–C) Wilcoxon matched-pairs signed rank tests were performed to compare values with or without colchicine/nocodazole. Two-tailed p values: (B) **p = 0.0078, ***p < 0.001; (C) ***p < 0.001, **p = 0.002; (D) ordinary one-way ANOVA with Holm-Sidak’s correction for multiple tests was performed. *p = 0.015, ***p < 0.001. (E) Ordinary one way ANOVA with Sidak’s correction for multiple tests was performed. *p = 0.011, ***p < 0.001.

Activation of the pyrin inflammasome in response to RhoA-inhibiting toxins depends on microtubule network integrity and is inhibited by microtubule-depolymerizing drugs (e.g., colchicine or nocodazole) (Gao et al., 2016; Van Gorp et al., 2016). Similarly, colchicine and nocodazole inhibited pyroptosis induced by steroid catabolites in primary human monocytes (Figures 4A and 4B). Colchicine also reduced IL-1β release in response to etiocholanolone and pregnanolone (Figure 4C), while it had no detectable action on NLRP3-dependent pyroptosis. Interestingly, the observed inhibition was only partial in most donors (Figures 4B and 4C) and was lost at higher doses of pregnanolone (Figure S4A). These results suggest that these high steroid catabolite concentrations can overcome the microtubule integrity requirement to activate the pyrin inflammasome in monocytes.

Current knowledge places the activation of the pyrin inflammasome downstream of RhoA GTPase inhibition (Akula et al., 2016; Xu et al., 2014). Yet, we observed no decrease in RhoA activity after etiocholanolone, a result that contrasted with the robust RhoA inhibition observed upon TcdA treatment (Figures 4D and S4B). This observation suggests that steroid catabolites activate pyrin by a mechanism distinct from the one triggered by TcdA. Accordingly, low doses of steroid catabolites synergized with suboptimal doses of TcdA to promote cell death (Figures S4C and S4D) and IL-18 release (Figure S4E).

We then investigated the pyrin domains required for steroid catabolite-mediated pyroptosis and IL-1β release. Pyrin proteins lacking either the pyrin (PYD) domain, the exon 2-encoded phosphorylated linker (PLD), the B-box, the coiled-coil, or the B30.2 domains were stably expressed in U937 cells (Figure S4F). The resulting cell lines were treated with pregnanolone, etiocholanolone, or TcdA. While the B30.2 domain was dispensable for TcdA-mediated response, steroid catabolites did not trigger release of IL-1β in the absence of the B30.2 domain (Figure 4E). Conversely, in the absence of the phosphorylated linker domain (PLD), steroid catabolites triggered IL-1β release, further strengthening the evidence that the activation mechanism is primarily independent of step 1. The PYD, B-box, and coiled-coil domains were required for both steroid catabolites and TcdA responses. Furthermore, all the different cell lines responded similarly to NLRP3 stimulation by LPS + nigericin.

The difference in the B30.2 dependence was further validated by comparing pyrin serine 242 dephosphorylation after treatment with TcdA or steroid catabolites. Indeed, TcdA and UCN-01 triggered the dephosphorylation of both WT and ΔB30.2 pyrin proteins, while etiocholanolone and pregnanolone only triggered pyrin dephosphorylation in the presence of the B30.2 domain (Figure 4F). These experiments indicated that pyrin dephosphorylation happened in a B30.2-dependent manner specifically after addition of steroid catabolites.

Although the exact molecular mechanisms remain to be deciphered, these experiments revealed an activation mechanism that strongly differs from the one triggered by RhoA-inhibiting toxins. Indeed, pyrin activation by steroid catabolites is initiated in a B30.2-dependent manner, takes place in the absence of RhoA inhibition, and does not require the PLD, which includes the two serine residues required to launch TcdA/B-mediated pyrin responses (Gao et al., 2016).

### The response to steroid catabolites is specific to human pyrin

**Figure 5.**
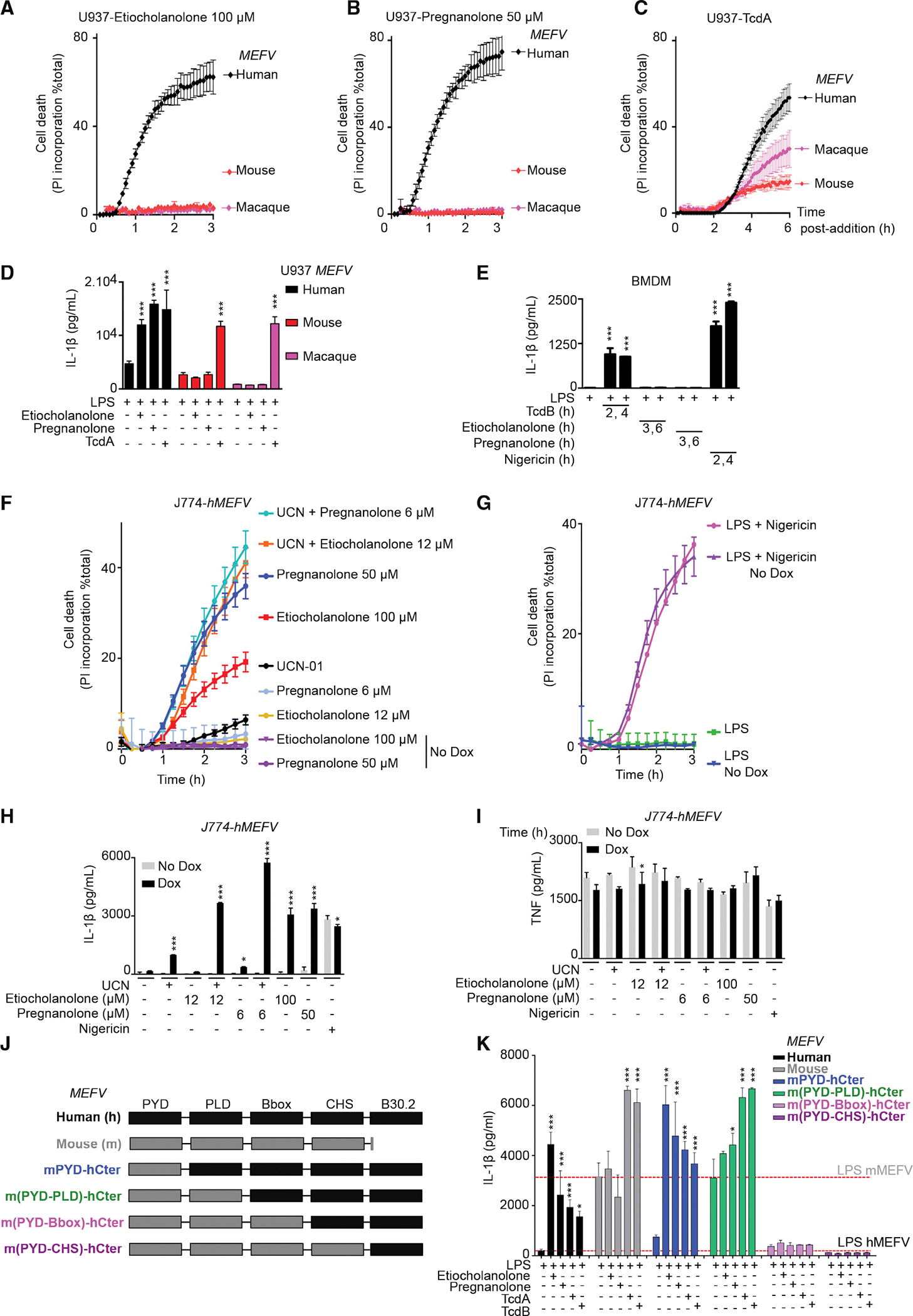
The human specificity of the response to steroid catabolites is intrinsic to the pyrin protein (A–C) Doxycycline-induced U937 monocytes or (D and K) PMA-differentiated U937 macrophages expressing human (black), mouse (red), *Macaca fascicularis* (magenta) or the indicated chimeric (J and K) *MEFV* were treated with the indicated stimuli. (A–C) Propidium iodide (PI) incorporation was monitored every 5 min for 3–6 h. (D and K) IL-1β concentration in the supernatant was quantified at 3 h (etiocholanolone, pregnanolone) or 6 h (TcdA, TcdB) post-addition. (E) WT bone marrow-derived macrophages (BMDM) were primed for 16 h with LPS (100 ng/mL) and treated with TcdB (10 ng/mL), etiocholanolone (100 μM), pregnanolone (50 μM), or nigericin (10 μg/mL). IL-1β concentration in the supernatant was quantified at the indicated time point post-compound addition. (F–I) J774 macrophages expressing or not (Dox or No Dox) human *MEFV* were treatedwith the indicated stimuli. (F and G) PI incorporation was monitored every 5 min for 3 h. (H and I) Cells were primed for 3 h with LPS before stimuli addition. (H) IL-1β and (I) TNF concentrations in the supernatant were quantified at 3 h post-addition. (K) The dotted lines indicate the basal value in LPS-treated U937 cells expressing human *MEFV* or murine *MEFV*. (A–I and K) One experiment representative of three independent experiments with mean and SEM of biological triplicates is shown. (D and E, H, I, and K) One-way ANOVA with Sidak’s test was used, ***p < 0.001 (H), *p = 0.214 (I), *p = 0.0304 (K), from left to right: *p = 0.0123, p = 0.0159.

Mouse pyrin does not contain a B30.2 domain (Chae et al., 2000) but is a functional protein triggering inflammasome activation in response to RhoA-inhibiting toxins (Xu et al., 2014). In addition, the B30.2 domain is highly polymorphic in primates (Schaner et al., 2001). Since we observed a total dependence on the B30.2 domain for the response to steroid catabolites, we assessed whether pyrin proteins from other species could promote responsiveness to steroid catabolites. Etiocholanolone and pregnanolone did not trigger pyroptosis in U937 cells expressing either mouse or macaque pyrin (Figures 5A, 5B, S5A, and S5B), while these cells underwent cell death in response to TcdA (Figure 5C). These results were further confirmed by quantifying IL-1β secretion (Figure 5D). The unresponsiveness of the murine pyrin inflammasome to etiocholanolone and pregnanolone was further validated in primary murine macrophages (Figure 5E).

To test whether the lack of response of murine macrophages to steroid catabolites was intrinsic to the pyrin protein or linked to a more general defect, we expressed human pyrin in the murine macrophage cell line, J774 (Figure S5B). The expression of human pyrin was sufficient to recapitulate the responses seen in human cells (Figures 5F and 5H). The response to NLRP3 stimulation was independent of human pyrin expression (Figures 5G and 5H) while, as expected, TNF levels were not impacted by human pyrin expression (Figure 5I). To map the species specificity in regard to pyrin domains, chimeric mouse-human *MEFV* constructs were generated and expressed in U937 cells (Figures 5J and S5C). Inclusion of the murine PYD domain did not impair the response to steroid catabolites, while inclusion of both murine PYD and PLD reduced but did not abolish the response to steroid catabolites (Figures 5K and S5D). The response to *C. difficile* toxins was not affected in either of the two chimera (Figure 5K). Chimeras with larger murine N-terminal pyrin were not functional, possibly due to low expression level (Figure S5C). Altogether, these results demonstrate that the response to steroid catabolites is human specific and that this species specificity is conferred, in an intrinsic manner, by the pyrin C-terminal domains.

### Monocytes from FMF patients display a moderate increase in the response to steroid catabolites compared with HDs

**Figure 6.**
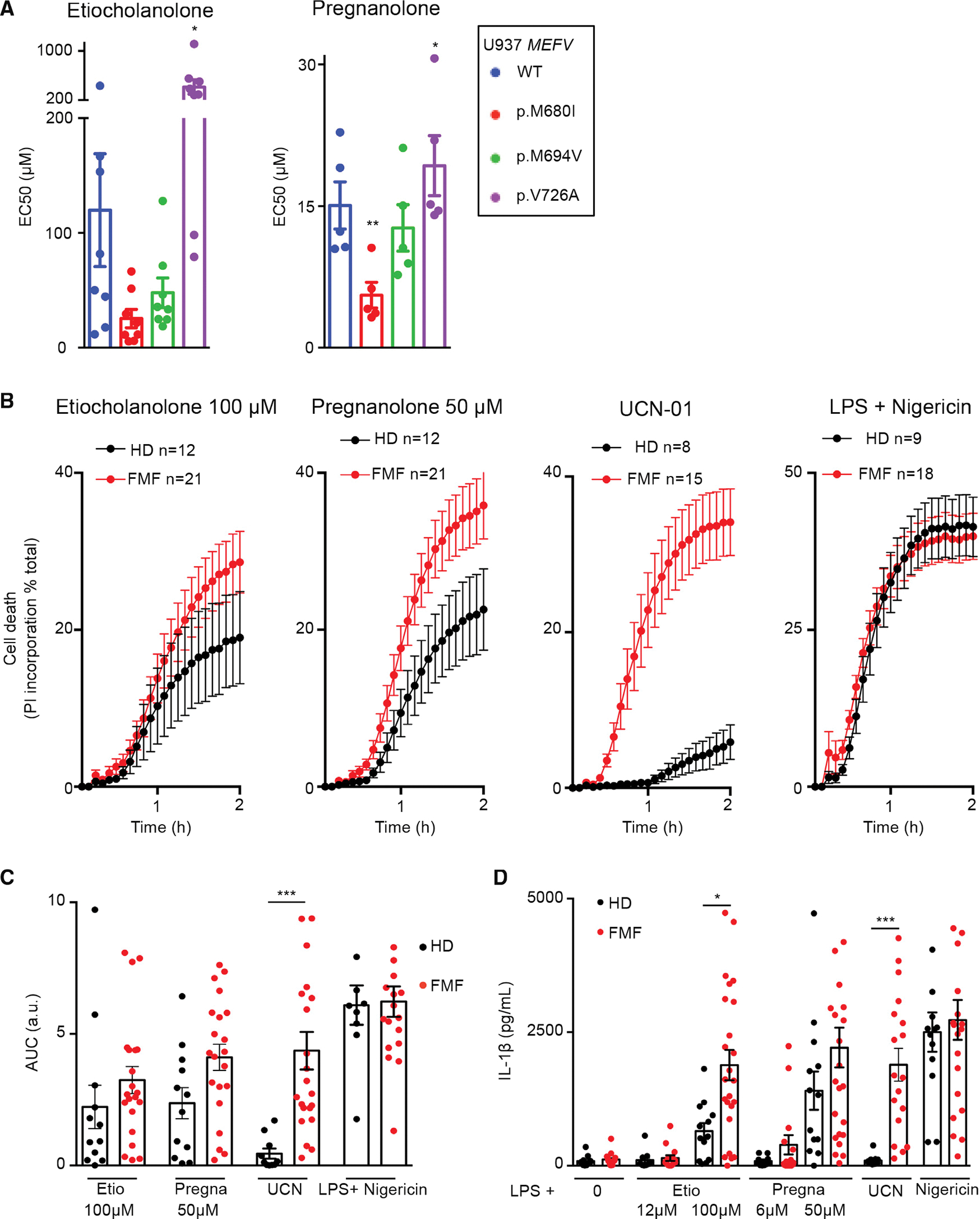
Monocytes from FMF patients display a moderately increased response to steroid catabolites compared with HDs (A) U937 expressing the indicated *MEFV* variant were exposed to the indicated steroid catabolite and the EC_50_ was determined at 3 h post-addition. (B) Monocytes from HD (n = 8–12) or FMF patients (n = 15–21) were treated with the indicated stimuli. Propidium iodide (PI) incorporation was monitored every 5 min for 2 h. (C) The corresponding area under the curve (AUC) are shown. (D) IL-1β concentrations were determined at 3 h post addition of the indicated molecules. (A) Mean and SEM of five to eight independent experiments are shown. Each dot represents the mean value of a triplicate from one experiment. RM one-way ANOVA with Dunnet’s multiple comparisons test was performed. Etio *p = 0.018; Pregna *p = 0.033 **p = 0.0042. (B) Mean and SEM of 8–21 individuals are show as indicated, each one corresponding to the average of a biological triplicate. (C) Each dot corresponds to the mean AUC of one individual performed in triplicates, the bar represents the mean of 8–21 individuals. (D) Each dot corresponds to the mean IL-1β concentrations of one individual performed in triplicates, the bar represents the mean of 8–21 individuals. (C and D) One-way ANOVA with Sidak’s multiple comparison test was applied; *p = 0.015, ***p < 0.001.

We then investigated whether FMF-associated mutations in *MEFV* exon 10 (altering the B30.2 domain) had an impact on the response of the pyrin inflammasome to steroid catabolites. U937 cells expressing three clearly pathogenic *MEFV* variants (Touitou, 2001) were exposed to etiocholanolone and pregnanolone (Figures 6A and S6A). While p.M680I and p.M694V mutations decreased these EC_50_ values (reaching statistical significance for p.M680I in the case of pregnanolone), the less severe p.V726A mutation (Cekin et al., 2017) significantly increased the EC_50_ of both etiocholanolone and pregnanolone. We then evaluated the steroid catabolites response of primary monocytes from FMF patients presenting at least one p.M680I or p.M694V mutation. A trend toward a faster and stronger cell death response was observed in response to steroid catabolites in monocytes from FMF patients compared with HDs (Figures 6B and 6C). No difference was observed in response to NLRP3 inflammasome engagement (Figures 6B and 6C) while, as described previously (Magnotti et al., 2019, 2020), UCN-01-mediated pyroptosis was specifically observed in monocytes from FMF patients (Figures 6B and 6C).

FMF monocytes released on average 4.5- and 1.6-fold more IL-1β than HD monocytes in response to low or high concentrations of pregnanolone, respectively (Figure 6D). In response to 100 μM etiocholanolone, monocytes from FMF patients released significantly more IL-1β than HD monocytes (p = 0.015). The differences in IL-1β concentrations were much stronger in response to UCN-01 (19.9-fold increase, p < 0.001), while no difference was observed upon NLRP3 stimulation. Altogether, these results suggest that FMF patients display a moderate increase in steroid catabolite-induced inflammasome responses that could contribute to inflammatory flares and be dependent on the *MEFV* genotype.

The pyrin inflammasome in FMF patients is mostly controlled at the step1 level (pyrin dephosphorylation) (Magnotti et al., 2019), likely explaining why the response to steroid catabolites (acting primarily on the step 2) is not drastically affected by FMF-associated *MEFV* mutations. We then decided to investigate the effects of steroid catabolites on cells from PAAND patients, in which the pyrin inflammasome step1 is constitutively activated leading to a pyrin inflammasome controlled mostly at step 2.

### Monocytes from PAAND patients respond to low concentrations of steroid catabolites in the absence of step 1 activator

**Figure 7.**
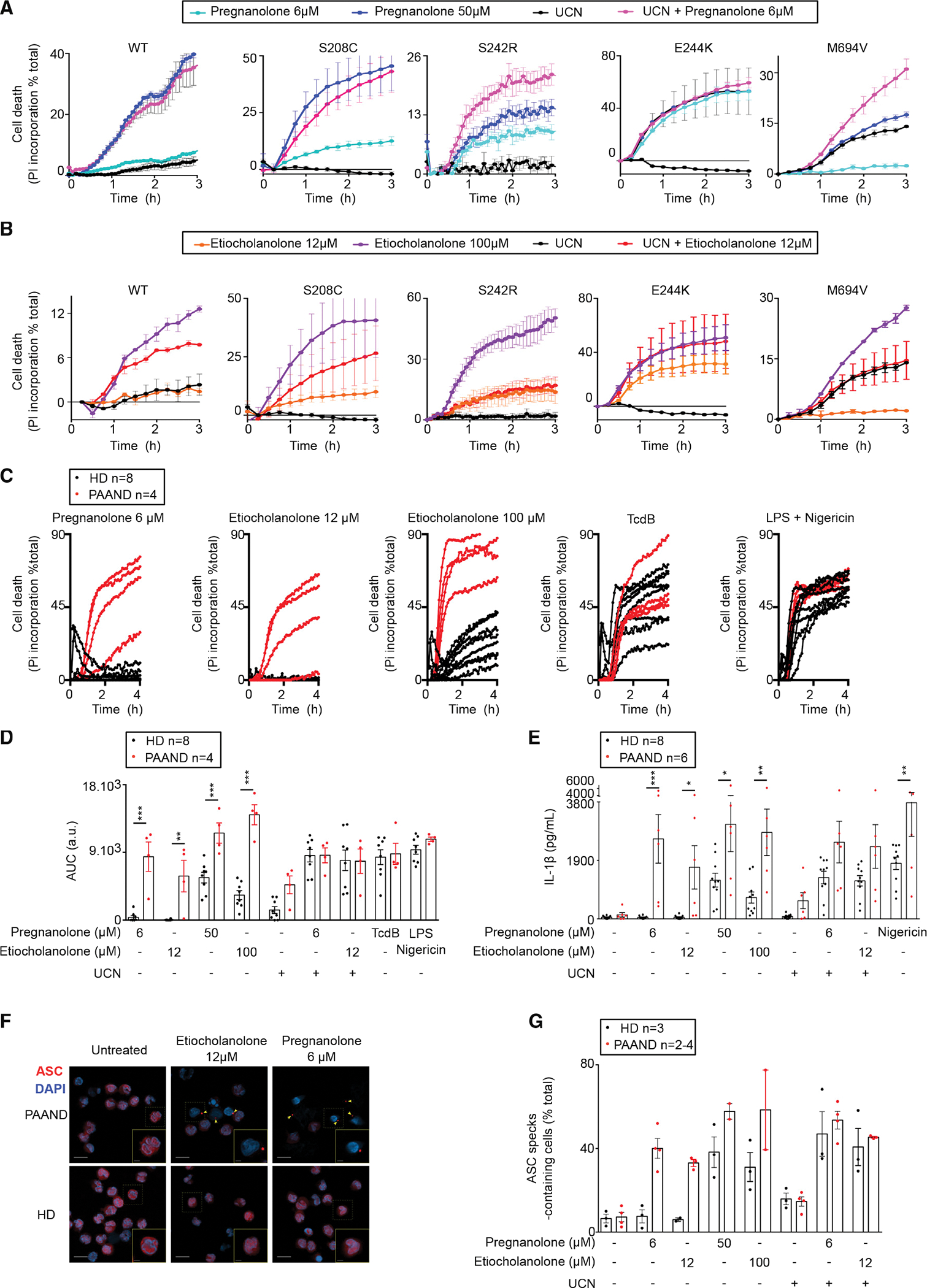
PAAND mutations confer hyper-responsiveness to steroid catabolites (A and B) U937 cell lines expressing WT or the indicated PAAND MEFV variant were treated with the indicated stimuli. Propidium iodide (PI) incorporation was monitored every 15 min for 3 h. (C–G) Monocytes from PAAND patient(s) (red, n = 4–6) or HDs (black, n = 8) were treated with the indicated stimuli. (C) PI incorporation was monitored every 15 min for 4 h. (D) The corresponding area under the curve (AUC)s are shown. (E) IL-1β concentrations were determined in the cell supernatant after 3 h of LPS treatment followed by 1.5 h of the indicated treatment. (F and G) ASC speck formation (indicated by yellow arrows) was monitored by immunofluorescence at 90 min post-treatment. (F) Representative images are shown. Scale bars, 10 μM (main pictures) and 2.5 μM (insets). (G) Quantification is shown. (A and B) One experiment representative of three independent experiments is shown. (A and B) Mean and SEM or (C) mean of triplicates are shown. (D and E) Each dot represents the mean of a triplicate for one individual. The bar represents the mean ± SEM. One way ANOVA with Sidak’s correction for multiple test was performed. (D) **p = 0.0011, ***p < 0.001; a.u., arbitrary units. (E) Etio 12 μM *p = 0.043; Pregna 50 μM *p = 0.016; Etio 100 μM **p = 0.0026; LPS Nig **p = 0.0064, ***p = 0.0001. (G) Kruskal-Wallis test with Dunn’s multiple comparison was performed. Each dot corresponds to the percentage of ASC specks based on more than 100 cells counted per condition. Each dot represents the value for one individual. The bar represents the mean ± SEM.

To assess the impact of PAAND-associated *MEFV* mutations on the response to steroid catabolites, we generated U937 cells expressing either of three reported PAAND mutations (p.S208C, p.S242R, and p.E244K; Figure S7A). Contrary to WT or p.M694V-expressing cell lines, all PAAND cell lines died quickly in response to low concentrations of pregnanolone or etiocholanolone (Figures 7A and 7B). UCN-01 synergized with the low concentrations of steroid catabolites in p.S208C-expressing cells, whereas it had no additional effect in p.S242R- or p.E244K-expressing cells. Phosphorylation of serine residue 242 and interaction of 14–3-3 chaperone with the neighboring residues may thus be more important to maintain pyrin inactive than phosphorylation of serine residue 208. This hypothesis is consistent with the fact that p.S242R and p.E244K mutations promote disease in a dominant manner while p.S208C does so in a recessive manner (Hong et al., 2019; Masters et al., 2016; Moghaddas et al., 2017).

We then investigated four distinct exon 2-encoded *MEFV* mutations identified in patients, and that have been either assigned as “variant of unknown significance” (p.E148Q, p.G250A) or likely pathogenic (p.E167D, p.T267I) and associated with a clinical FMF-like phenotype. Interestingly, one mutation (p.G250A), absent from Gnomad, gave a partial response to low doses of steroid catabolites suggesting it may affect pyrin step1 and be a pathogenic PAAND-like mutation. None of the other three mutations gave cellular phenotypes differing from WT pyrin-expressing cells (Figures S7B and S7C) suggesting that they correspond either to *MEFV* benign polymorphisms or to FMF-like mutations (which was not tested here).

We then assessed pyroptosis and IL-1β release in primary monocytes from PAAND patients from two independent families with heterozygous p.S242R mutation. At low concentration, pregnanolone and etiocholanolone triggered cell death (Figures 7C and 7D), IL-1β release (Figures 7D and 7E), and ASC speck formation (Figures 7F and 7G). In contrast, none of these responses were observed in monocytes from HDs. No differences were observed upon stimulation with TcdB or UCN-01 (in the presence or absence of low concentrations of steroid catabolites) (Figures 7C–7E and 7G). High concentrations of pregnanolone or etiocholanolone also triggered higher IL-1β release in PAAND patient monocytes compared with HD monocytes (Figure 7E), possibly due to a faster and stronger inflammasome response (Figures 7D and S7D).

These results thus demonstrate that PAAND patient monocytes strongly respond to low doses of steroid catabolites, suggesting that these molecules could contribute to inflammation in these patients and also to the distinct clinical features observed in PAAND and FMF patients.

## DISCUSSION

The identification of steroid catabolites as molecules triggering pyrin step 2 provides insights into pyrin activation mechanisms. First, it confirms that the two steps can be activated independently. Indeed, low doses of sex steroid catabolites do not impact pyrin phosphorylation, but the same doses trigger inflammasome activation in the presence of pyrin variants impaired for phosphorylation. Furthermore, step 2 is dependent on the B30.2 domain, and independent of PLD, while the inverse applies to TcdA-mediated pyrin activation. Finally, a coupling mechanism likely exists between the two steps since high doses of steroid catabolites trigger the full activation of pyrin. This coupling mechanism might be mediated by pyrin conformation changes affecting the phosphorylation/dephosphorylation balance mediated by PKN1/2 and by the calyculin A-sensitive phosphatase(s) (Malik et al., 2022). Alternatively, high doses of steroid catabolites might affect the activity of PKN1/2 or of the pyrin phosphatase(s). Of note, in this particular setting, what we initially termed “second step” is likely to be upstream of pyrin dephosphorylation.

In contrast to the TcdA/B responses, the response to steroid catabolites is human specific. In mice, the lack of the B30.2 domain partly explains the absence of response. Yet, we did not observe any response in bone marrow-derived macrophages from knockin mice presenting the murine pyrin protein fused to the human B30.2 domain (Chae et al., 2011; Park et al., 2020) (Figure S5E). The resulting chimeric pyrin does not include the human coiled-coil domain, likely explaining the lack of response of these cells. Indeed, the coiled-coil domain is required for steroid catabolite-mediated pyrin activation and is highly divergent between murine and human pyrin proteins (Figure S5A). Surprisingly, the expression of *Macaca fascicularis* pyrin did not confer responsiveness to steroid catabolites either, despite the presence of similar coiled-coil and B30.2 domains. *MEFV* has been subjected to a strong evolutionary pressure in primates (Schaner et al., 2001), possibly explaining the difference in steroid catabolite responsiveness between *M. fascicularis* and *Homo sapiens*.

Sex hormones are known to regulate immune responses, and their variations during menstrual cycle and pregnancy correlate with profound modifications in local and systemic immune responses (Beagley and Gockel, 2003; Stelzer et al., 2021; Regan et al., 2013). Particularly, IL-1β and IL-18 levels fluctuate during menstrual cycle and pregnancy (Azlan et al., 2020; Cannon and Dinarello, 1985; Romero et al., 2018). Since pregnanolone sensitizes cells to low doses of the bacterial toxin TcdA (Figures S4C–S4E), the increase in pregnanolone may lower pyrin inflammasome threshold toward the end of pregnancy and during menstruation. To our knowledge, plasma etiocholanolone/pregnanolone levels never reach micromolar concentrations (pregnanolone plasma level can reach up to 70 nM during pregnancy [Deligiannidis et al., 2016; Hill et al., 2007]), so it is unlikely that physiological steroid catabolites concentrations could fully activate the pyrin inflammasome in the absence of a synergistic signal or pathogenic *MEFV* mutations. Interestingly, flares in women with FMF are frequently associated with menstruation (Akar et al., 2006; Duzgun et al., 2006; Kishida et al., 2020), which correspond to the peak of progesterone catabolism. While this correlation is appealing, demonstrating the pathophysiological role of pregnanolone (and/or etiocholanolone) remains challenging, especially due to the current lack of animal models and the complexity of the metabolome changes during menstrual cycles and pregnancy (Stelzer et al., 2021).

Progesterone and testosterone were completely inactive with regard to pyrin inflammasome activation. Similarly, all the modifications tested on the etiocholanolone or the pregnanolone backbones decreased inflammasome responses. We thus believe that human pyrin has specifically evolved in humans to sense these catabolites. Interestingly, Alimov and colleagues identified a synthetic molecule, BAA473, that shares the same steroid backbone and the same stereochemistry as etiocholanolone and pregnanolone and activates pyrin. In contrast to the molecules identified here, which are endogenous molecules, the relevance of BAA473 remains to be established, although, theoretically, BAA473 could be generated from the secondary bile acid, deoxycholic acid (Alimov et al., 2019). BAA473-mediated pyrin inflammasome activation likely proceeds through an identical mechanism as steroid catabolites.

In addition to being sex hormone catabolites, pregnanolone and etiocholanolone are also neurosteroids that can be generated de novo in the central nervous system (Yilmaz et al., 2019). Neurosteroid levels vary greatly, depending on the specific physiological situations, and can reach submicromolar to micromolar concentrations (Hosie et al., 2006). Particularly, pregnanolone levels increase during psychological stress (Park et al., 2017; Ströhle et al., 2002), a condition known to promote flares in FMF patients (Kishida et al., 2020). Whether etiocholanolone or pregnanolone could locally reach high enough concentrations to prime or activate the WT pyrin inflammasome in a particular neurological environment (and contribute to neuroinflammation) is unclear at the moment. Interestingly, we observed that at least one *MEFV* mutation (p.P373L) confers responsiveness to nanomolar concentrations of steroid catabolites (Figure S6B) indicating that physiological concentrations can modulate pyrin inflammasome activation. All other neurosteroids tested (pregnenolone and pregnenolone sulfate) were inactive, and we could not identify receptors upstream of the pyrin inflammasome to explain the pyroptotic effect of pregnanolone and or etiocholanolone. The pyrin B30.2 domain contains a hydrophobic pocket that has been hypothesized to bind a ligand. Similarly, the butyrophilin 3A1 B30.2 domain displays a pocket that accommodates microbialderived phosphoantigens resulting in the activation of gamma delta T cells (Sandstrom et al., 2014). It is thus tempting to speculate that pregnanolone and etiocholanolone could directly bind the pyrin B30.2 domain to activate the inflammasome. This speculation is supported by the impact of some B30.2-affecting mutations (e.g., V726A) which decreased the efficacy of steroid catabolites to activate the pyrin inflammasome (Figure 6A).

Importantly, experiments performed in the late 1950s have demonstrated the fast and potent pyrogenic activity of steroids of endogenous origin, including etiocholanolone and pregnanolone, upon injection in human volunteers (Kappas et al., 1957, 1960). These historical experiments thus validate these steroid catabolites as potent *in vivo* inflammation inducers, and our results strongly suggest that activation of the pyrin inflammasome was at the origin of this enigmatic “steroid fever” (Kappas et al., 1961).

### Limitations of the study

The requirement for the B30.2 domain and the lack of observed RhoA inhibition suggest that steroid catabolites activate pyrin by an unconventional mechanism. This conclusion is based on negative data (lack of observed RhoA inhibition) and chimeric or truncated pyrin variants for which it is difficult to exclude non-specific impact on the overall protein structure and function. We speculate that the pyrin B30.2 may directly bind steroid catabolites to promote pyrin activation step 2 although, so far, we did not manage to demonstrate this interaction.

While the steroid fever experiments indicate that these molecules can trigger inflammation upon injection in humans and could be used as proinflammatory mediators, we were unable to test whether physiological steroid catabolites concentrations can locally prime or fully activate the WT pyrin inflammasome. At the moment, most of the experiments were performed with steroid catabolites concentrations ≈100-fold higher than those found in serum. Novel animal models and/or clinical studies are needed to investigate the links between pyrin inflammasome and steroid hormones catabolism at key life stages in females and males.

## STAR★METHODS

### RESOURCE AVAILABILITY

#### Lead contact

Further information and requests for resources and reagents should be directed to the lead contact, Thomas Henry (thomas.henry@inserm.fr).

#### Materials availability

The WT, p.M694V (FMF), p.S08C/S242R (PAAND) pyrin-encoding plasmids have been previously deposited to Addgene (ID 134702, 703, 706). Other point mutant plasmids are available upon request to the lead contact. All unique/sable reagents generated in this study are available from the lead contact without restriction.

#### Data and code availability

- Raw data (full Western blot images and Raw data) have been deposited at Mendeley and are publicly available as of the date of publication. DOI is listed in the key resources table.
- This paper does not report original code
- Any additional information required to reanalyze the data reported in this paper is available from the lead contact upon request.

### EXPERIMENTAL MODEL AND SUBJECT DETAILS

#### Human subjects

The study was approved by the French Comité de Protection des Personnes SUD-EST IV (CPP,#L16–189), Ile de France IV (CPP, #2018/95) and by the French Comité Consultatif sur le Traitement de l’Information en matière de Recherche dans le domaine de la Santé (CCTIRS, #16.864) and the Leuven/Onderzoek Ethic committee (#S58600). The authors observed a strict accordance to the Helsinki Declaration guidelines. HD blood was provided by the Etablissement Français du Sang in the framework of the convention #14–1820. Informed consent was obtained from all healthy donors and patients.

All FMF patients fulfilled the Tel Hashomer criteria for FMF, had at least one mutation in the *MEFV* gene and are listed in Table S2. PAAND patients all bear heterozygous p.S242R mutation. Three patients have been previously reported (Van Nieuwenhove et al., 2021) while three patients (1 adult 2 children) were identified by Pr. Tran (CHU Nîmes). The potential carriage of *MEFV* mutations in HD was not assessed. Blood samples from HD were drawn on the same day as patients.

The age and sex of patients are provided in Table S2. The number of patients in each experiment is reported in the corresponding figure panels.

#### Cell lines

The human myeloid cell line U937 (ATCC #CRL-1593.2) was grown in RPMI 1640 medium with glutaMAX-I supplemented with 10% (vol/vol) FCS, 100 IU/mL penicillin, 100 μg/mL streptomycin (ThermoFischer Scientific) at 37°C. U937 cell line was derived in 1974 from malignant cells obtained from the pleural effusion of a 37-year-old, White, male patient with histiocytic lymphoma. U937 cells were obtained from CelluloNet-BRC, SFR Bioscience, tested mycoplasma-free. The cell line has not been authenticated.

293T cell line (ATCC #CRL-3216) is an epithelial-like cell line that was isolated from the kidney of a female fetus. 293T cells were grown in DMEM medium with glutaMAX-I supplemented with 10% (vol/vol) FCS at 37°C. 293T cells were obtained from Anira-vectorology platform, SFR Bioscience, tested mycoplasma-free. The cell line has not been authenticated.

#### Primary cells

Bone-marrow progenitors from the femurs and tibias from female 6–12 weeks-old C57BL6/J (Charles River) or *MEFV*^M694VKI^ (Chae et al., 2011) mice were obtained in the framework of the ethical approval ENS_2012_061 (CECCAPP, Lyon, France). Mice were maintained in the PBES animal facility by trained staff with daily monitoring of animal behavior and husbandry conditions in agreement with the French laws (“Décret n 2013–118 du 1er février 2013 relatif à la protection des animaux utilisés à des fins scientifiques”). Cages contained enrichment and bedding material. Water and food was given ad libitum. Progenitors were differentiated into bone-marrow derived macrophages (BMDMs) during 6 days in DMEM medium with glutaMAX-I supplemented with 10% (vol/vol) FCS and 10% M-CSF-containing supernatant at 37°C. Progenitors were seeded in non-tissue culture treated petri dishes and following differentiation were lifted in PBS without calcium and magnesium using cell scrapers (Sarstedt).

Human monocytes, neutrophils, lymphocytes: Blood was drawn in heparin-coated tubes and kept at room temperature overnight. The age and sex of patients is provided in Table S2, the age and sex of healthy donors was not available due to full anonymization of donors from the EFS blood bank. Monocytes were isolated as previously reported (Magnotti et al., 2019). Briefly, peripheral blood mononuclear cells (PBMCs) and neutrophils were isolated by density-gradient centrifugation. Monocytes were further isolated by magnetic positive selection using CD14 MicroBeads (Miltenyi Biotec) following manufacturer’s instructions. Lymphocytes were recovered from the negative fraction of monocytes isolation. Neutrophils were separated from red blood cells (RBCs) using Dextran (Sigma, #31392), residual RBCs were lysed with ice-cold bidistilled water and contaminating CD14^+^ monocytes were excluded using CD14 MicroBeads. Live cells were enumerated by flow cytometry (BD Accuri C6 Flow Cytometer®). All human cells were grown in RPMI 1640 medium with glutaMAX-I supplemented with 10% (vol/vol) FCS, 100 IU/mL penicillin, 100 μg/mL streptomycin (ThermoFischer Scientific) at 37°C in the presence of 5% CO_2_.

### METHOD DETAILS

#### Compound library and reagents

The Prestwick® Chemical Library (Prestwick Chemical, Illkirch, France) amounts to a total of 1,199 compounds arrayed in fifteen 96-well plates. This 2013 version of the library contains mostly US Food and Drug Administration (FDA)-approved drugs. All the compounds were stored in DMSO at −20°C. Mother plates 1–14 were at a concentration of 2 mg/mL, which corresponds to 6.32 ± 2.8 mM, and the last one at a concentration of 10 mM. All the compounds and their final concentrations are listed in Table S1. Etiocholanolone (3α-hydroxy-5β-androstan-17-one, #R278572), Testosterone (#86500), Androsterone (3α-hydroxy-5α -androstan-17-one, #31579), 3β -hydroxy-5β-androstan-17-one (R213691), Progesterone (#P8783), Pregnanolone (5-beta-pregnan-3α-ol, 20-one, #P8129), cortisol (#C-106), UCN-01 (#U6508), Doxycycline (#D9891) were from Sigma. Pregnanolone (5-beta-pregnan3α-ol, 20-one, #P8150–000), 11-one: (5-beta-pregnan-3α-ol-11, 20-dione, #P7850–000), 11αOH: (5β-pregnan-3α, 11α-diol-20-one, #P6400–00), 11βOH: (5-β-pregnan-3-α, 11β-diol-20-one, #P6420–000), Hemisuccinate: (5-β-pregnan-3-α, 21-diol-20-one, 21 hemisuccinate, #P6944–000), 21OH: (5-β-pregnan-3-α, 21-diol-20-one, #P6920–000), 17OH (5-β-pregnan-3-α, 17 diol-20-one, #P6570–000), 20H (5-β-pregnan-3-α-ol, #P7800–000), Sulfate (5β-pregnan-3α-ol-20-one sulfate, sodium salt, #P8168–000) were from Steraloids. Tetrahydrocortisol (#T293370) was from Toronto Research Chemicals. LPS-EB Ultrapure (#tlrl-3pelps), Nigericin (#tlrl-nig) were from Invivogen. TcdB was from Abcam (#ab124001). TcdA was purified from *Clostridium difficile* VPI10463 strain, as previously described (von Eichel-Streiber et al., 1987; Popoff, 1987).

#### Chemical library screening

All robotic steps were performed on a Tecan Freedom EVO platform. Compounds from the Prestwick Chemical Library® were evaluated at a 1:1,000 or 1:2,000 dilution of the original stock for plates 1–14 and plate 15, respectively (see Table S1). 1 μL of DMSO solutions was spiked into dry well of F-bottom clear cell culture treated 96-wells plates (Greiner Bio One), with columns 1 and 12 devoted to controls and used to calculate the Z′-factor. U937 cells expressing p.S242R *MEFV* were treated or not (counterscreen) with doxycycline (1 μg/mL) for 16 h, centrifuged and seeded at 10^5^ cells per well (100μL final volume) in RPMI 1640 without phenol red, 10% FCS, 1mM HEPES, 1% PSA, 1mM Glutamine, in the presence of propidium iodide at 5 μg/mL. Following 90 min of incubation at 37°C, fluorescence intensity (excitation wavelength at 535 nm and emission wavelength at 635 nm) corresponding to propidium iodide incorporation was measured on a microplate reader (Infinite M1000, Tecan). The average Z′ value was 0.67 ± 0.12, indicating a robust and reliable assay. Mean fluorescence + 3SD was retained as a threshold. Compounds triggering cell death only in the presence of doxycycline were considered as pyrin-specific and defined as hits.

#### Genetic manipulation

*Casp1*^KO^ and *GSDMD*^KO^ cell lines, U937 cell lines expressing WT, p.S208C, p.S242R, p.M694V, p.M680I under the control of a doxycycline-inducible promoter, have been previously described (Lagrange et al., 2018; Magnotti et al., 2019). p.[V726A], p.[E244K], ΔPYD, ΔPLD, ΔB-Box, ΔCoiled-coil, ΔB30.2 *MEFV* were generated by mutagenesis of the pENTR1A-3xFlag *MEFV* using primers presented in Table S3, pfu ultra II Fusion high fidelity polymerase (Agilent) followed by digestion of the parental plasmid using Dpn1 restriction enzyme. The resulting plasmids were validated by sequencing and the mutated *MEFV* constructs were transferred into the GFP-expressing plasmid pINDUCER21 (Meerbrey et al., 2011) under the control of a doxycycline-inducible promoter using LR recombinase (Invitrogen). Lentiviral particles were produced in 293T cells using pMD2.G and psPAX2 (from Didier Trono, Addgene plasmids #12259 and #12260), and pINDUCER-21 plasmids. U937 cells were transduced by spinoculation and selected at day 4 post-transduction based on GFP expression on an Aria cell sorter and maintained polyclonal. Pyrin expression was induced by treatment with doxycycline (1 μg.mL^−1^) for 16 h before stimulation. All parental cell lines were tested for mycoplasma contamination.

#### Inflammasome activation

For cytokine quantification, primary monocytes were seeded in 96-well plates at 5 × 10^3^ cells/well, in RPMI 1640, GlutaMAX medium (Thermofisher) supplemented with 10% fetal calf serum (Lonza) and incubated for 3 h in the presence of LPS (10 ng/mL, Invivogen). Primary monocytes were then treated for 1 h 30 with nigericin (5 μM, Invivogen); UCN-01 (12.5 μM, Sigma), TcdB (125 ng/mL, Abcam) or steroid catabolites at the indicated concentrations. When indicated, monocytes were treated with colchicine (1 μM, Sigma), nocodazole (5 μM, Sigma), VX-765 (25 μM, Invivogen), MCC950 (10 μM, Adipogen AG-CR1–3615) or Calyculin A (Sigma, 208851) 30 min before addition of steroid catabolites, UCN-01, TcdB or Nigericin. Following the incubation, cells were centrifuged, and supernatants were collected.

To assess cytokine release, 8 × 10^4^ U937 cells per well of a 96 wells plate were exposed to 100 ng.mL^−1^ of phorbol 12-myristate 13-acetate (PMA; InvivoGen) for 48 h and primed with LPS at 50 ng/mL for 3 h. When applicable, nigericin was used at 50 μg.mL^−1^. Supernatant was collected at 3 h post treatment. Levels of IL-1β, IL-18 or TNF in cell supernatants were quantified by ELISA (R&D Systems). The number of replicates and independent experiments are listed in the corresponding figure legends.

#### ASC specks immunofluorescence

Monocytes were fixed with paraformaldehyde 2% for 20 min before spreading onto poly-lysine adhesion slides (Thermo Scientific^™^) using the Cytospin3 (Shandon) 5 min at 450 rpm. Following permeabilization with Triton X-100 (0.1% in PBS), cells were stained using anti-ASC (Santa Cruz, sc22514R, 4 μg.mL^−1^), Alexa 594-goat anti rabbit antibodies (Invitrogen, A-110088, 10 μg.mL^−1^) and DAPI (100 ng.mL^−1^). ASC specks were visualized on the Zeiss LSM800 confocal microscope. Quantification was performed on 10 fields per sample.

#### Real time cell death and EC50 calculation

For real time cell death assays, monocytes and U937 cells were seeded at 2 or 5 × 10^4^ per well of a black 96 well plate (Costar, Corning), respectively, in the presence of propidium iodide (PI, Sigma) at 5 μg/mL. Three technical replicates per conditions were done. Real time PI incorporation was measured every 5 to 15 min immediately post-stimuli addition on a fluorimeter (Tecan) using the following wavelengths: excitation 535 nm (bandwidth 15 nm); emission 635 nm (bandwidth 15 nm) (Case and Roy, 2011; Pierini et al., 2012). Cell death was normalized using PI incorporation in cells treated with Triton X100 for 15 min (=100% cell death) and PI incorporation at each time point in untreated cells (0% cell death). As a further correction, the first time point of the kinetics was set to 0. The areas under the curve were computed using the trapezoid rule (Prism 6; GraphPad). To calculate the EC50 (Half maximal effective concentration), the normalized cell death at 3 h post-compound addition was used. To compare different cell lines, butyrate (1mM) was added for 16 h (in the meantime as doxycycline) to revert transgene silencing (Chen et al., 1997). The different concentrations were log-transformed, and a non-linear regression was applied using the Log (agonist) vs. normalized response-variable slope model (Prism 6; GraphPad). The least squares (ordinary) fitting method was applied.

#### RhoA activity

RhoA activity was determined by G-LISA (Cytoskeleton) following manufacturer’s instructions.

#### Crosslinking, immunoprecipitation, immunoblot

Cells were lysed in 25mM Tris HCl, 150mM NaCl, 1mM EDTA and 0.1% NP-40 buffer containing Mini Protease Inhibitor Mixture (Roche) and sodium fluoride (Sigma) by a quick freezing and thawing step. Flag-Pyrin was immuno-precipitated using anti Flag M2 affinity gel (Sigma). ASC was cross-linked in the insoluble pellet using DSS (Disuccinimidyl suberate, ThermoFisher #21655) 2 mM (1 h at 37°C). Proteins were separated by SDS/PAGE on precast 4–15% acrylamide gels (Bio-rad) and transferred to TransBlot® Turbo^™^ Midi-size PVDF membranes (Bio-rad). Antibodies used were mouse monoclonal anti-FLAG® (Sigma-Aldrich, clone M2; 1:1,000 dilution), anti-Pyrin (Adipogen, AL196, 1: 1,000 dilution), anti-phospho S242 Pyrin (Abcam, ab200420; 1:1,000 dilution) (Gao et al., 2016), anti-human Caspase-1 (Santa Cruz, sc515, 1: 1,000 dilution), anti-human GSGMD (sigma, HPA044487, 1: 1,000 dilution), anti-human IL-1β (Cell signaling, #12703, 1: 1,000 dilution), anti-ASC (Santa Cruz, sc22514R, 1:1,000 dilution). Cell lysates were reprobed with a mouse monoclonal antibody anti-β-actin (clone C4, Millipore; 1:5,000 dilution).

### QUANTIFICATION AND STATISTICAL ANALYSIS

Normality was verified using D’Agostino & Person omnibus normality test, Shapiro-Wilk normality test or Kolmogorov-Smirnov test with Dallal-Wilkinson-Lille for p value if the number of values was too small for the former test. Gaussian distribution was assumed for technical triplicates. Unmatched normalized values were analyzed by Ordinary one-way ANOVA with Sidak’s multiple comparisons test. When normality could not be verified, matched values were analyzed by the Friedman test, with Dunn’s correction or using Sidak’s multiple comparisons test. Normal matched values were analyzed with RM one-way ANOVA, with the Greenhouse-Geisser correction and Dunnett correction for multiple comparisons. Unmatched values, for which normality could not be verified, were analyzed using Kruskal-Wallis analysis with Dunn’s correction. Effect of treatment was analyzed by Wilcoxon matched-pairs signed rank tests. Prism 7 (GraphPad) was used for statistical analyses. The statistical analyses and parameters for each experiments are listed in the corresponding figure legends.

## Supplementary Material

1

2

## Figures and Tables

**KEY RESOURCES TABLE:** 

REAGENT or RESOURCE	SOURCE	IDENTIFIER

Antibodies		

ASC	Santa Cruz	Cat#sc22514R; RRID: AB_2174874
AlexaFluor594-Goat anti-rabbit	Invitrogen	A-110088
Flag	Sigma	M2 clone; RRID: AB_439698
Pyrin	Adipogen	Cat#AL196;RRID: AB_2490454
Phospho-S242-Pyrin	Abcam	Cat#ab200420; RRID: AB_2922814
Caspase-1	Santa cruz	Cat#sc515;RRID: AB_630975
GasderminD	Sigma	Cat#HPA044487;RRID: AB_2678957
IL-1b	Cell signaling Technology	Cat#12703;RRID: AB_2737350
Actin	Millipore	Clone C4;RRID: AB_2223041

Bacterial and virus strains		

Clostridioides difficile	Michel Popoff (Pasteur institute)	strain VPI10463

Biological samples		

CD14+ monocytes	Healthy donors, FMF patients, PAAND patients from EFS or clinical centers	Anonymous
Neutrophils	healthy donors from EFS	Anonymous

Chemicals, peptides, and recombinant proteins		

Etiocholanolone (3α-hydroxy-5β-androstan-17-one)	Sigma	R278572
Testosterone	Sigma	86500
Androsterone (3α-hydroxy-5α-androstan-17-one)	Sigma	31579
Progesterone	Sigma	P8783
Pregnanolone (5-beta-pregnan-3α-ol, 20-one)	Sigma	P8129
5-beta-pregnan-3α-ol-11,20-dione	Steraloids	P7850-000
5-β-pregnan-3-α, 11 β-diol-20-one	Steraloids	P6420-000
5-β-pregnan-3-α, 21-diol-20-one, 21 hemisuccinate	Steraloids	P6944-000
5-β-pregnan-3-α, 21-diol-20-one	Steraloids	P6920-000
5-β-pregnan-3-α, 17 diol-20-one	Steraloids	P6570-000
5-β-pregnan-3-α-ol	Steraloids	P7800-000
5β-pregnan-3α-ol-20-one sulfate, sodium salt	Steraloids	P8168-000
Tetrahydrocortisol	Toronto Research Chemicals	T293370
LPS-EB Ultrapure	Invivogen	tlrl-3pelps
Nigericin	Invivogen	tlrl-nig
TcdB	Abcam	ab124001, Uniprot P18177
TcdA	Purified from Clostridium difficile VPI10463 strain by M. Popoff	Uniprot P16154
Pregnanolone	Steraloids	P8150-000
Cortisol	Sigma	C-106
Doxycycline	Sigma	D-9891
UCN-01	Sigma	U6508
Dextran	Sigma	31392

Prestwick chemical library	See Table S2	“FDA-approved library” 2013 version

Deposited data		

Raw data and full Western Blot	Mendeley	https://doi.org/10.17632/7pfjtn7xhv.1

Experimental models: Cell lines		

U937	CelluloNet-BRC, SFR Bioscience	ATCC #CRL-1593.2
293T	Anira-vectorology platform, SFR Bioscience	ATCC #CRL-3216

Experimental models: Organisms/strains		

Bone marrow derived macrophages	Mouse: C57BL/6J (JAX® Mice Strain)	Charles River #632

Oligonucleotides		

See Table S3	This Paper	N/A

Recombinant DNA		

pMD2.G	Addgene (Didier Trono)	12259
psPAX2	Addgene (Didier Trono)	12260
pENTR1A-3xFlag-MEFV	Addgene (Thomas Henry)	167018
p21-V726A-MEFV	This study	Available upon request
p21-E244K-MEFV	This study	available upon request
p21-ΔPYD-MEFV	This study	available upon request
p21-ΔPLD-MEFV	This study	available upon request
p21-ΔB-Box-MEFV	This study	available upon request
p21-ΔCoiled-coil-MEFV	This study	available upon request
p21-ΔB30.2-MEFV	This study	available upon request

Software and algorithms		

Prism	GraphPad	Version 6.0h

## Highlights

- A chemical screen identifies sex hormone catabolites as specific pyrin activator
- The pyrin activation mechanism is human specific and B30.2 dependent
- Patients with specific mutations in pyrin are highly responsive to the catabolites
- This sterile inflammatory activity likely explains the experimental steroid fever
