# Supplementary material for: Steroid hormone catabolites activate the pyrin inflammasome through a non-canonical mechanism
Source: Cell Rep. Author manuscript; Available in PMC 2022 Nov 2. (PMC9626387; doi:10.1016/j.celrep.2022.111472)
Supplement: 1 [file NIHMS1843395-supplement-1.pdf]

Cell Reports, Volume *41*

Supplemental information

Steroid hormone catabolites activate the pyrin inflammasome through a non-canonical mechanism

Flora Magnotti, Daria Chirita, Sarah Dalmon, Amandine Martin, Pauline Bronnec, Jeremy Sousa, Olivier Helynck, Wonyong Lee, Daniel L. Kastner, Jae Jin Chae, Michael F. McDermott, Alexandre Belot, Michel Popoff, Pascal Sève, Sophie Georgin-Lavialle, Hélène Munier-Lehmann, Tu Anh Tran, Ellen De Langhe, Carine Wouters, Yvan Jamilloux, and Thomas Henry

Name	Structure	LogP	Name	Structure	LogP
Etiocholanolone (3α-hydroxy 5β-androstan-17one)	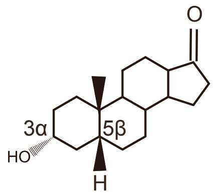	4.2	5β-Pregnan-3α,11α-diol -20-one	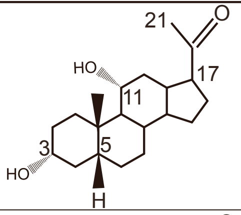	
Pregnanolone (3α-hydroxy 5β-Pregnan-20one)	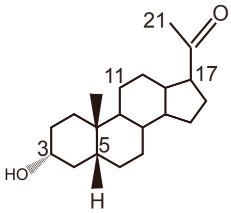	4.8	5β-Pregnan-3α,11β-diol -20-one	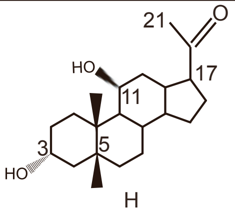	
Testosterone	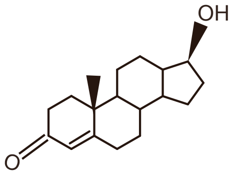	4.1	3α-hydroxy 5β-Pregnan-11,20-dione	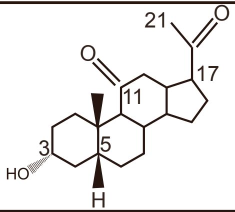	
Progesterone	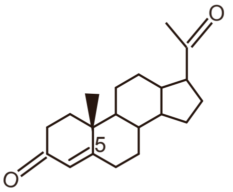	4.7	5β-Pregnan-3α, 17diol-20-one	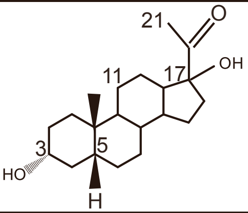	
Cortisol	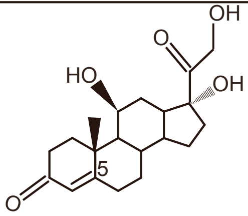	2.6	5β-Pregnan-3α-ol	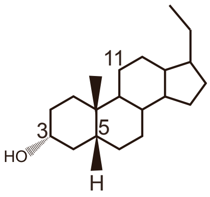	
Tetrahydro-cortisol	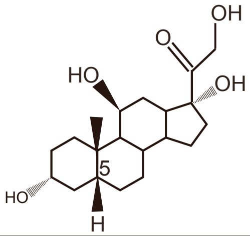	2.8	5β-Pregnan-3α,21-diol-20-one	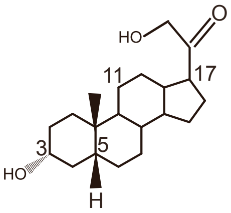	
Pregnanolone-sulfate	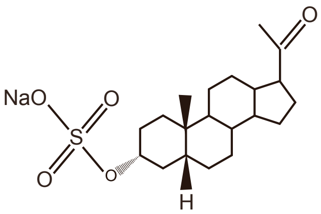	5.8	5β-Pregnan-3α,21-diol-20-one 21-hemisuccinate	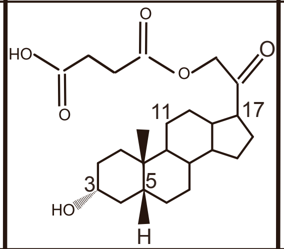	
3β-hydroxy 5β-androstan-17one	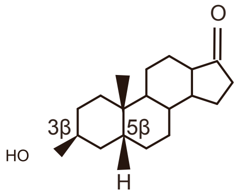		Androsterone	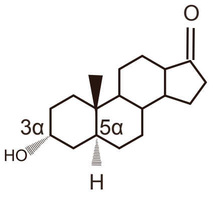	4.2

Figure S1: Structure of the steroid molecules tested in this study (related to Figure 2).

Whenever available, the lipophilic value (LogP) is indicated .

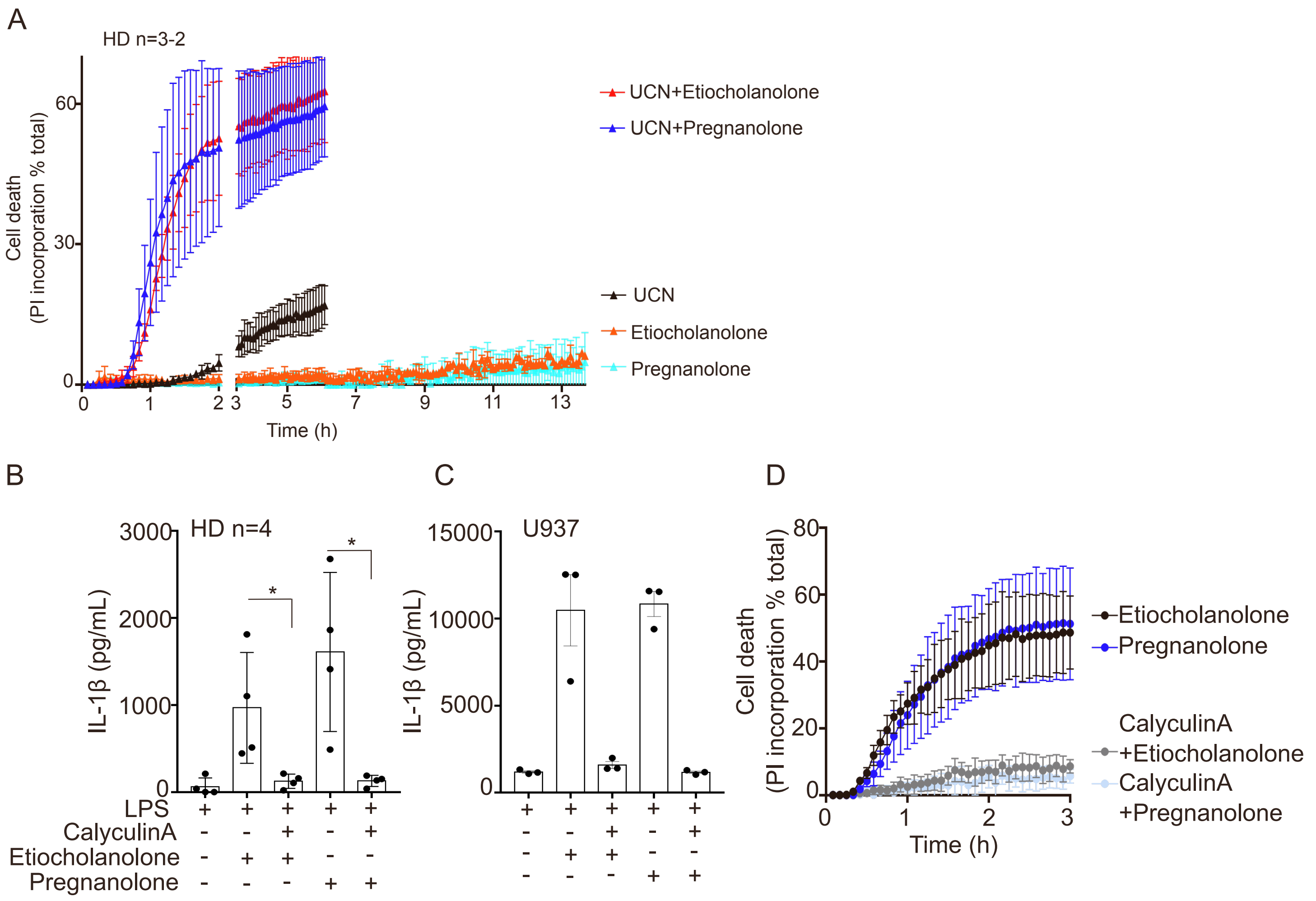

Figure S2: Long treatment with low concentrations of steroid catabolites, influence of the phosphatase inhibitor, Calyculin A- related to Figure 3.

(A) Primary monocytes from healthy donors (HD, n=3-2) were pre-treated with pregnanolone (6 µM) or etiocholanolone (12 µM) for 1 h followed by addition of UCN-01. Cell death was monitored every 5 min for 6-13 h. UCN-01 alone triggers delayed apoptosis while low concentrations of steroid catabolites are inactive in the absence of UCN-01. (B) Primary monocytes from healthy donors (HD, n=4) were primed 3 h with LPS (10 ng/ml), pre-treated with Calyculin A (40 nM) for 30 minutes and treated with etiocholanolone (100 µM) or pregnanolone (50 µM). IL-1β concentrations were determined in the cell supernatant 1 h 30 after the final treatment. (C) Doxycycline-induced, PMA-differentiated U937 macrophages expressing WT pyrin were primed 3 h with LPS (50 ng/ml), pre-treated with Calyculin A (40 nM) for 30 minutes and treated with etiocholanolone (100 µM) or pregnanolone (50 µM). IL-1β concentrations were determined in the cell supernatant 3 h after the final treatment. (D) U937 cells expressing WT pyrin (in the presence of doxycycline) were pre-treated with Calyculin A (40 nM) for 30 minutes and treated with etiocholanolone (100 µM) or pregnanolone (50 µM). Cell death was monitored by following propidium iodide incorporation every 5 min for 3 h. (B) each dot represents the value for one HD. Ordinary one way ANOVA with Holm-Sidak's multiple comparison test was performed. *p<0.005. (C-D) One experiment with technical triplicates representative of three independent experiments is shown.

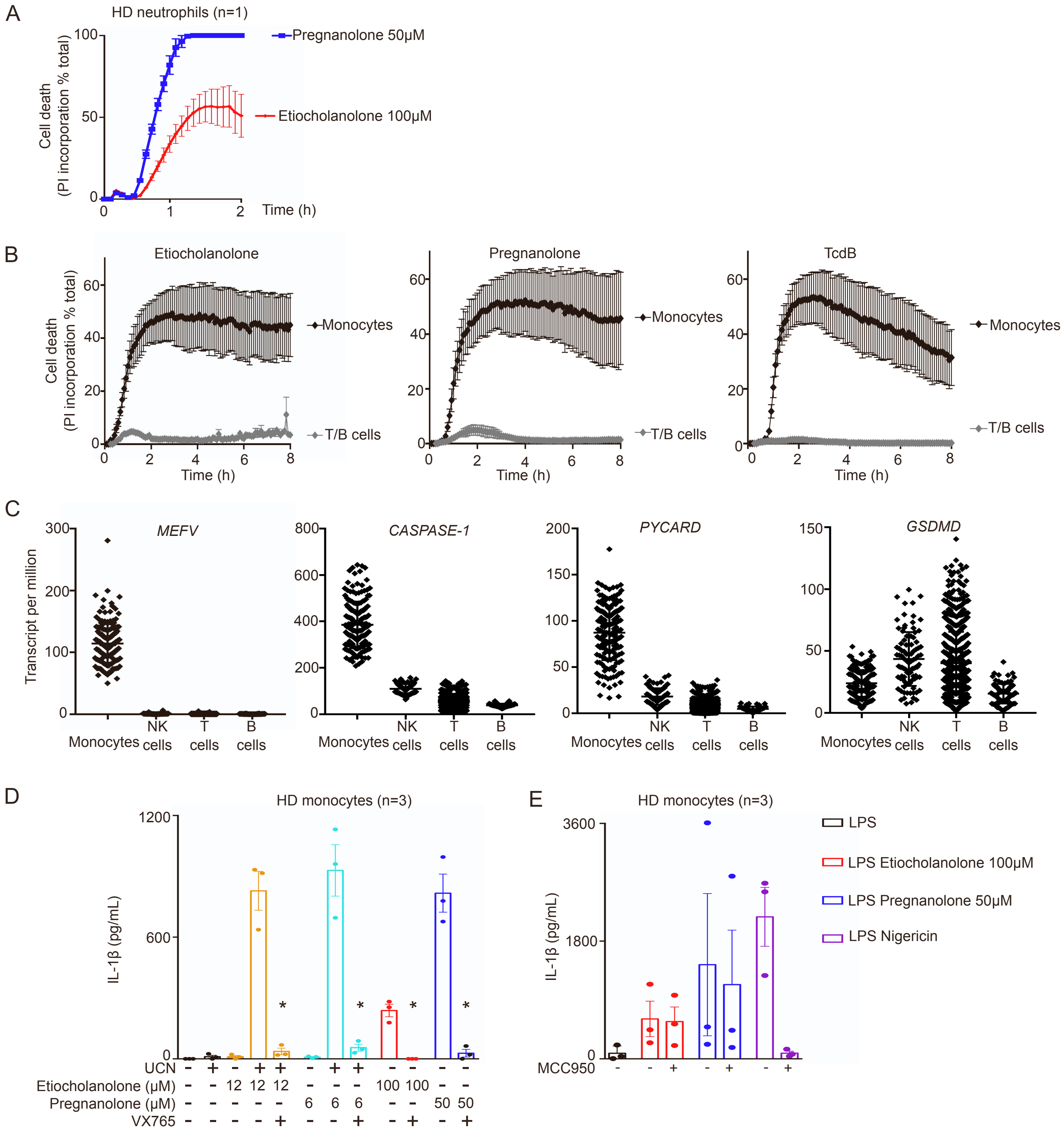

Figure S3: Responsiveness of different cell types and influence of the caspase-1 inhibitor, VX765 , and the NLRP3 inhibitor, MCC950- related to Figure 3.

(A) Propidium iodide incorporation in neutrophils from one HD treated with pregnanolone or etiocholanolone was monitored every 5 min for 2 h. (B) Primary monocytes and lymphocytes from healthy donors (n=3) were treated with etiocholanolone (100 μM), pregnanolone (50 μM) or TcdB (125 ng/ml). Cell death was monitored every 5 min for 8 h. (C) MEFV, CASPASE-1, PYCARD, GSDMD gene expression levels in monocytes, NK cells and lymphocytes as determined by RNAseq. (D-E) HD monocytes (n=3) were treated with LPS for 2 h 30 followed by addition or not of (D) the caspase-1 inhibitor VX-765, (E) the NLRP3 inhibitor (MCC950) and 30 min later of the indicated molecules. IL-1β concentrations were determined in the cell supernatant 1 h 30 after the final treatment. (A) One experiment representative of two independent experiments is shown. Mean and SEM of a technical triplicates are shown. Cell death was normalized using untreated neutrophils (0%) and Triton X100-treated neutrophils (100%). (B) each dot represents the mean of three healthy donors values each one from a technical triplicate. SEM is shown. (C) Each dot represents one RNAseq value from one healthy donor extracted from the database of Immune Cell Expression: https://dice-database.org/). (D-E): Each dot represents the value of one HD (mean of a triplicate). The bar represents the mean +/- SEM of 3 HD values. Matched one-way ANOVA with Sidak's multiple comparisons test was performed to compare untreated vs. VX-765-treated samples. *: $p<0.05$.

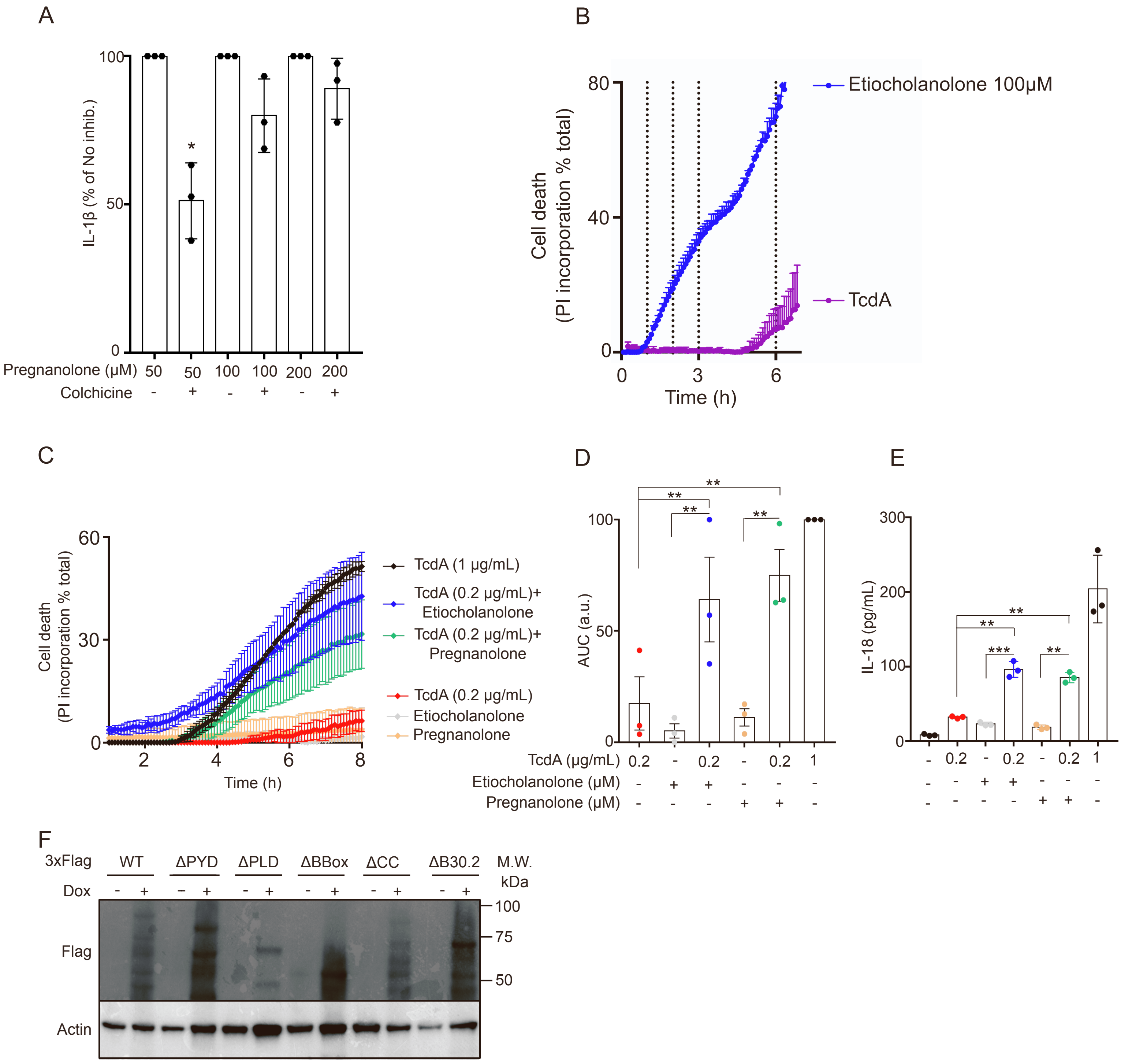

Figure S4: Increasing pregnanolone doses overcome colchicine inhibition and low concentrations of catabolites synergize with low TcdA doses for pyrin inflammasome activation (related to Figure 4).

(A) Monocytes from HD (n=3) were treated with colchicine and 30 min later with Pregnanolone (50-100-200 µM). IL-1β concentration in the supernatant was quantified at 3 h post-addition. Values obtained in the presence of inhibitors were normalized to the value obtained without the inhibitor. (B) U937 cells expressing WT pyrin (in the presence of doxycycline) were treated with etiocholanolone or TcdA. Cell death was monitored by following propidium iodide incorporation every 5 min for 7 h. The dotted vertical lines indicate the time points at which U937 cells were collected in a parallel experiment to assess RhoA inhibition (see Fig. 4D; 1, 2, 3 h for etiocholanolone and 3, 6 h for TcdA treatment). (C-D) U937 cells expressing WT pyrin (in the presence of doxycycline) were pre-treated with etiocholanolone or pregnanolone for 1h and treated with TcdA at the indicated concentrations. Cell death was monitored by following propidium iodide incorporation every 5 min for 8 h. (D) The Area Under the Curve (AUC) was computed for 3 different experiments. (E) Doxycycline-induced, U937 monocytes expressing WT pyrin were treated as in (C-D). IL-18 concentration in the supernatant was quantified at 6 h post-treatment. (F) U937 cell lines expressing the indicated 3xFlag-MEFV variants were analysed by Western blot in the presence or absence of doxycycline (Dox). Actin was used as a loading control. (A) One experiment performed with 3 HD is shown. Each dot represents the mean value from three biological replicates from 1HD. The bar represents the mean +/- SD of the 3 HD mean values. (B) Mean and SD from three biological replicates from one experiment (see Fig. 4D). (C-E) Each dot represents the mean value +/- SEM from three biological replicates. One experiment representative of two independent experiments is shown.

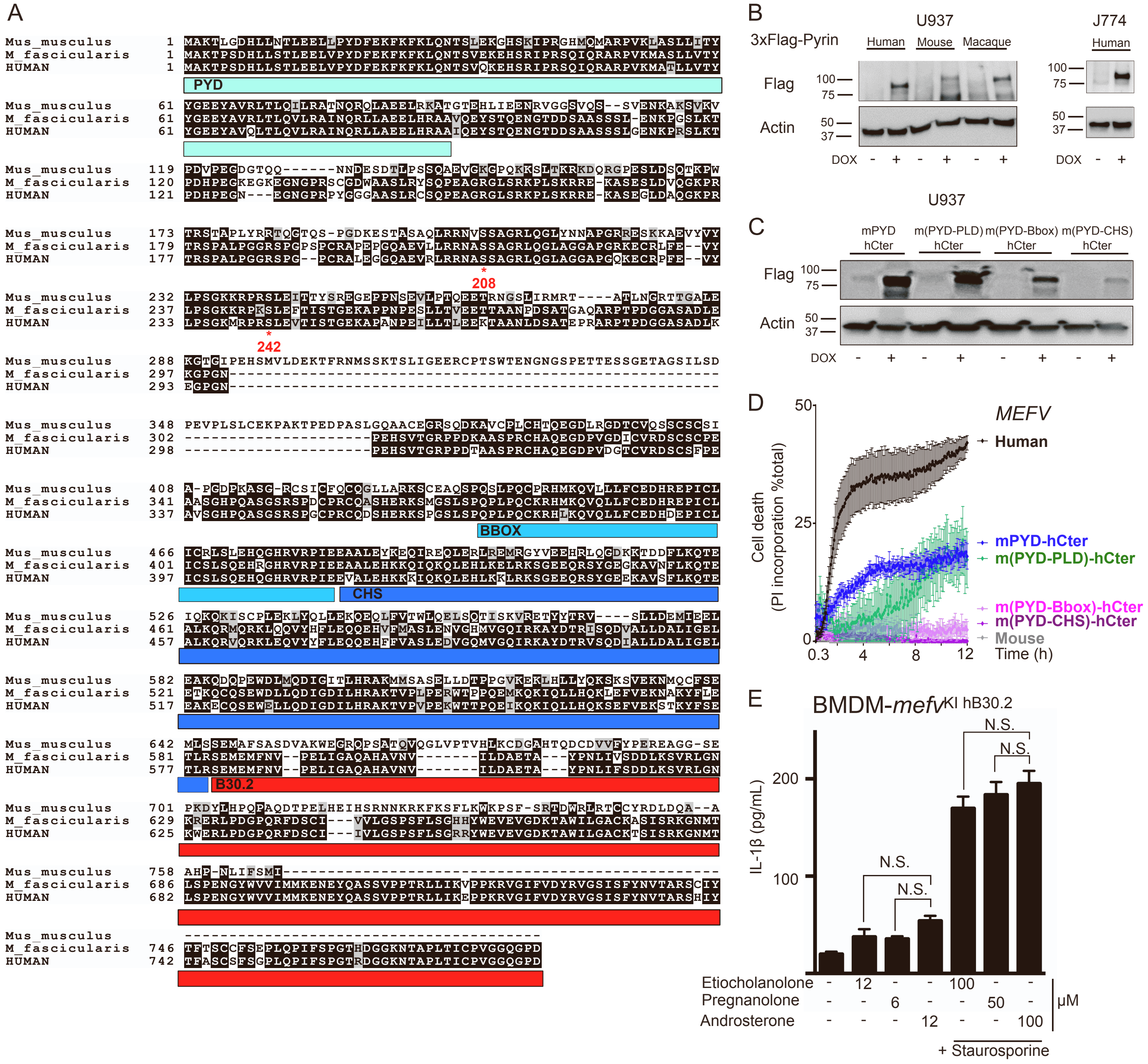

Figure S5 : Alignment of human, mouse and macaca fascicularis pyrin proteins, validation of cell lines with ectopic expression of human, mouse, macaque or chimeric pyrin, response of bone marrow derived macrophages (BMDM) from human B30.2 knock-in mice -related to figure 5.

(A) The PYD, the two critical serine residues, the B-Box, Central Helical scaffold (CHS) and B-30.2 domains are shown. (B-C) Western blot analysis of U937 (left panel) or J774 (right panel) cells expressing 3xFlag-pyrin from the indicated species or the indicated chimeric proteins. Cells were treated or not with doxycycline (DOX) and cell lysates were analysed with anti-Flag (top panel) or anti-actin (bottom panel) antibodies. (D) U937 cell lines expressing the indicated chimera were treated with Etiocholanolone (100 μM). Propidium iodide (PI) incorporation was monitored every 5 min for 12 h. (E) Bone marrow derived macrophages (BMDM) from human B30.2 knock-in mice do not respond to steroid catabolites. BMDM from mefvKI hB30.2p.M694V mice harbouring human B30.2 domain in fusion with murine pyrin protein were treated with the indicated steroid molecules for 1 h followed by addition (or not) of staurosporine (1 μM) for 3 h. IL-1β concentrations were determined in the cell supernatant 4 h after steroid addition. Androsterone (Andro) was used as a negative control based on Fig. 2C results. Etiocholanolone and pregnanolone treatment did not differ from androsterone treatment in the presence or absence of the PKC superfamily inhibitor, staurosporine, used here to trigger pyrin step 1. (D) Each dot represents the mean +/- SD of a biological triplicate. One experiment representative of two independent is shown. (E) The bar represents the mean +/- SEM of a biological triplicate. One experiment representative of two independent is shown. One way ANOVA with Sidak's multiple comparisons test was performed. N.S.: not significant.

A

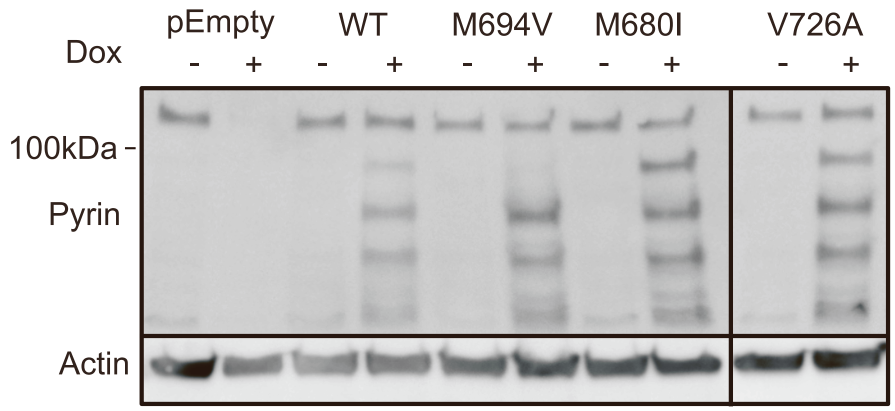

B

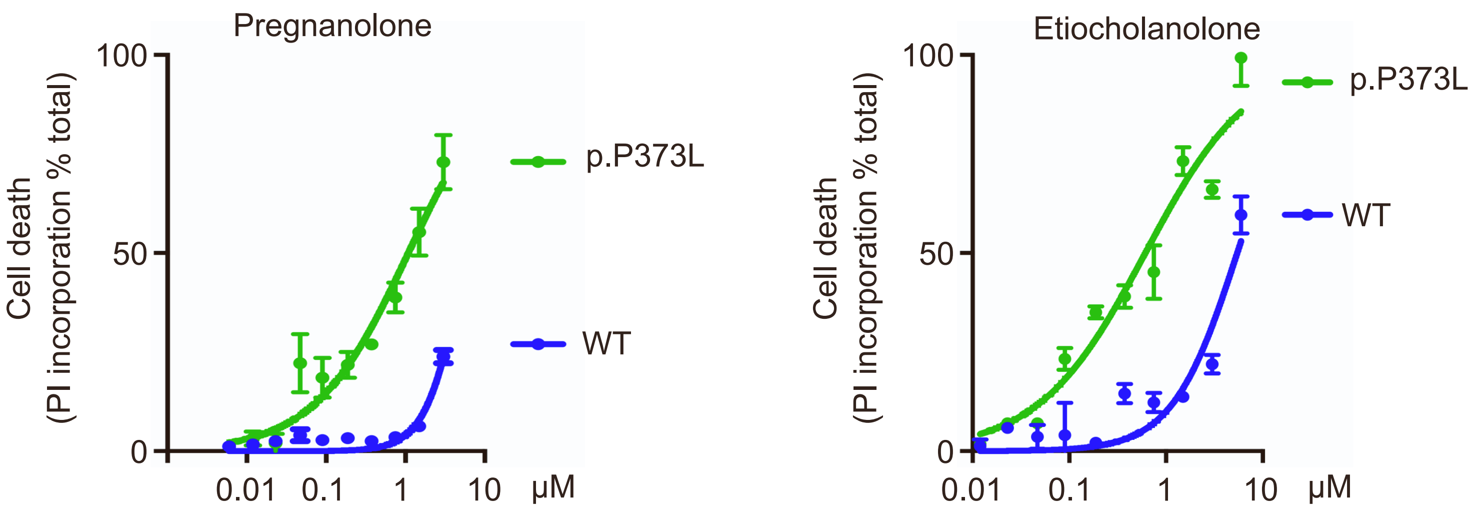

Supplemental Fig. S6: Western blot analysis of U937 cell expressing p.V726A pyrin and functional analysis of p.P373L MEFV variant-related to figure 6.

(A) U937 cell lines expressing the indicated pyrin variants were treated or not with doxycycline (Dox) and cell lysates were analysed with anti-pyrin (top panel) or anti-actin (bottom panel) antibodies. Cell lines expressing WT, M694V and M680I pyrin variants have been previously characterized. (B) p.P373L MEFV variant confers responsiveness to nanomolar concentrations of steroid catabolites.
U937 cells expressing the indicated MEFV variant (green p.P373L; blue WT) were treated with doxycycline for 16 h followed by addition of pregnanolone or etiocholanolone at various concentrations. Cell death was measured at 3 h post-addition. (B) One experiment representative of three independent experiment is shown. Mean and SEM of biological triplicates are shown. Non-linear regression curve computed using least squares fit method is shown.

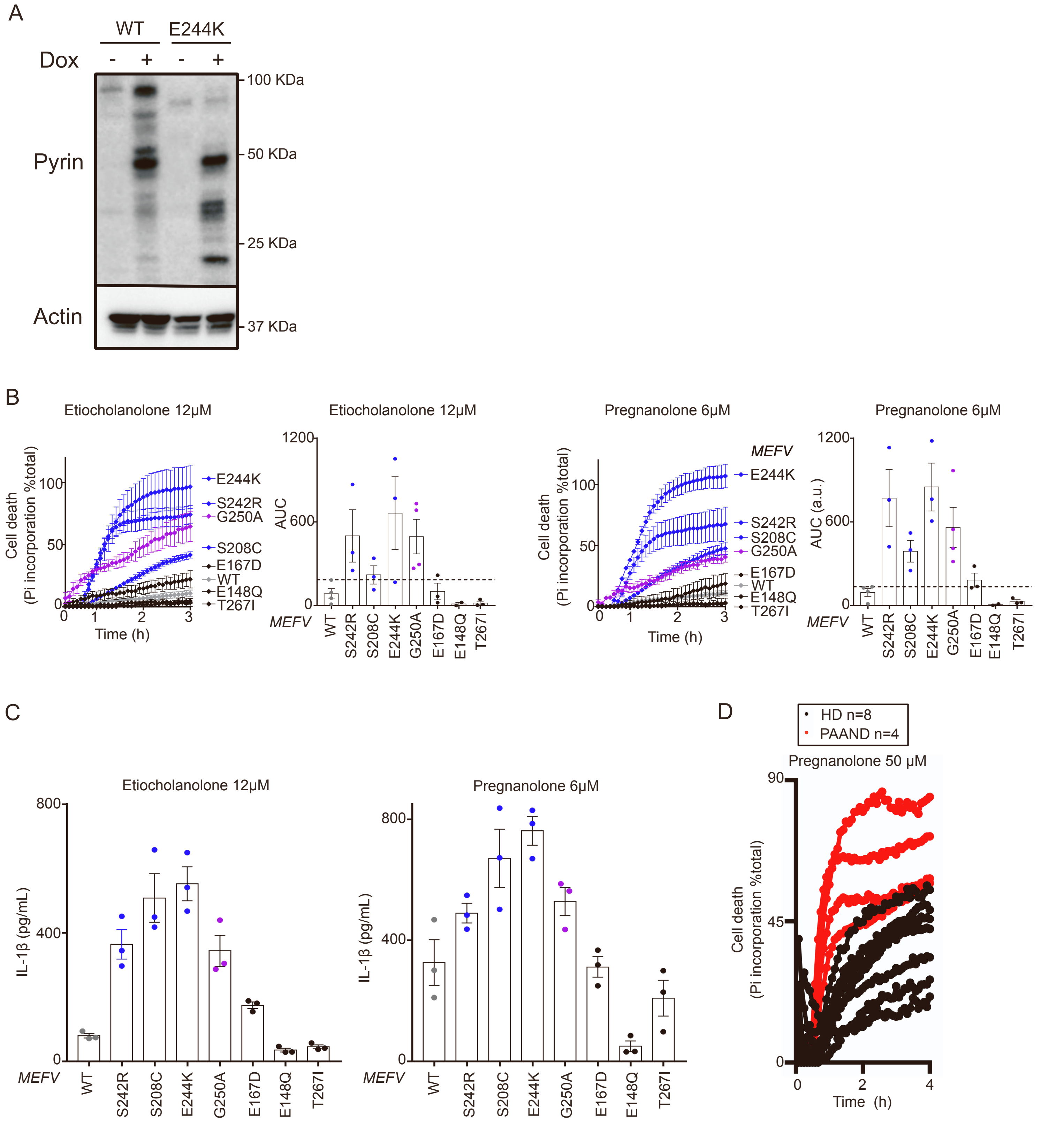

Figure S7:PAAND patients monocytes present a strong increase in steroid catabolites responses compared to HD-related to Figure 7.

(A) Western blot analysis of U937 cell expressing p.E244K pyrin variant. U937 cell lines expressing the indicated pyrin protein were treated or not with doxycycline (Dox) and cell lysates were analysed with anti-Pyrin (top panel) or anti-actin (bottom panel) antibodies. Cell lines expressing WT, S208C and S242R pyrin variants have been previously characterized 11. (B) U937 cells expressing MEFV exon 2 variants of unknown significance or WT MEFV were treated with low doses of etiocholanolone or pregnanolone. Cell death was monitored every 5 min for 3 h. (C) Doxycycline-induced, PMA-differentiated U937 macrophages expressing MEFV exon 2 variants of unknown significance or WT MEFV, were primed with LPS during 3 h and treated with low doses of etiocholanolone or pregnanolone. IL-1β concentration in the supernatant was quantified at 3 h post-treatment. (D) Monocytes from PAAND patients (red, n=4) or HD (black, n=8) were treated with pregnanolone (50 µM). Cell death/ propidium iodide incorporation was monitored in real time every 15 min for 4 h. (B) One real time cell death experiment representative from 3 independent experiments with each dot representing mean +/- SEM of a biological triplicate is shown. The Area Under the Curve (AUC) was computed for 3-4 independent experiments, each dot corresponds to the mean of three biological replicate from one experiment. The bar shows the mean +/- SEM from 3-4 independent experiments. (C) One experiment representative from 2 independent experiments is shown. Each dot represents the value of a single well, the bar represents the mean +/- SEM of a biological triplicate. (D) Each dot corresponds to the average of a triplicate for one individual.

Supplemental Table S2: List of patients-related to figures 6 and 7.

#	Sex (Male/Female)	Age (Years)	Disease	Genotype
1	M	72	FMF	M694V/M694V
2	M	34	FMF	M694V/M694V
3	M	20	FMF	M694V/M694V
4	M	22	FMF	M694V/M694V
5	F	45	FMF	M694V/M694V
6	M	45	FMF	M694V/M694V
7	M	21	FMF	M694V/M694V
8	F	47	FMF	M694V/M694V
9	F	36	FMF	M694V/M694V
10	M	56	FMF	M694V/M694V
11	M	28	FMF	M694V/M694V
12	M	20	FMF	M694V/M694V
13	F	52	FMF	M680I/V726A
14	M	40	FMF	M694V/V726A
15	F	22	FMF	M694V/V726A
16	M	32	FMF	M694V/I259V
17	M	26	FMF	M694del/0
18	M	33	FMF	M694V/0
19	F	24	FMF	M694V/0
20	M	20	FMF	M694V/0
21	M	49	FMF	M694V/0
22	F	44	FMF	M694V/0
23	F	42	FMF	M694V/0
24	M	57	PAAND	S242R/0
25	F	22	PAAND	S242R/0
26	M	78	PAAND	S242R/0
27	M	46	PAAND	S242R/0
28	M	13	PAAND	S242R/0
29	M	14	PAAND	S242R/0

Supplemental Table S3: Primers used in this study (related to STAR Methods)

Sequence (5'-3')	Used for
GAATATTCCACACAAGAAAACGGCACAGATG	ΔPYD
CGCGGCCGCAAGCTTGTC	ΔPYD
GAAGGCCACCAGACACGG	ΔPLD
CCTGAATGGCTGCCCTGT	ΔPLD
GAGGAGGTCGCCCTGGAA	ΔBBox
CTGGGGGCTTAGGCTTCC	ΔBBox
CGATCCTATGGGGAGGAG	ΔCcoil
CTTGTGTTCCAGGGCGAC	ΔCcoil
TTGAACATTTCCATTTCTTAACGCAGGGTTTCTGAGAAGTAC	ΔB30.2
GTACTTCTCAGAAACCCTGCGTTAAGAAATGGAAATGTTCAA	ΔB30.2
gatgcgacctagaagccttaaggtcaccatttcta	p.E244K
tagaaatggtgaccttaaggcttctaggtcgcatc	p.E244K
atcttcgtggactacagagctggaagcatctcctttta	p.V726A
taaaaggagatgcttccagctctgtagtccacgaagat	p.V726A
